# A genetically encoded sensor for in vivo imaging of orexin neuropeptides

**DOI:** 10.1038/s41592-021-01390-2

**Published:** 2022-02-10

**Authors:** Loïc Duffet, Seher Kosar, Mariangela Panniello, Bianca Viberti, Edward Bracey, Anna D. Zych, Arthur Radoux-Mergault, Xuehan Zhou, Jan Dernic, Luca Ravotto, Yuan-Chen Tsai, Marta Figueiredo, Shiva K. Tyagarajan, Bruno Weber, Miriam Stoeber, Nadine Gogolla, Markus H. Schmidt, Antoine R. Adamantidis, Tommaso Fellin, Denis Burdakov, Tommaso Patriarchi

**Affiliations:** 1 Institute of Pharmacology and Toxicology, University of Zurich, Zurich, Switzerland; 2 Department of Health Sciences and Technology, ETH Zurich, Zurich, Switzerland; 3 Optical Approaches to Brain Function Laboratory, Istituto Italiano di Tecnologia, Genova, Italy; 4 Center for Experimental Neurology (ZEN), Department of Neurology, Inselspital University Hospital Bern, University of Bern, Bern, Switzerland; 5 Department of Biomedical Research, University of Bern, Bern, Switzerland; 6 Circuits for Emotion Research Group, Max Planck Institute of Neurobiology, Martinsried, Germany; 7 International Max Planck Research School for Translational Psychiatry (IMPRS-TP), Munich, Germany; 8 Department of Cell Physiology and Metabolism, University of Geneva, Geneva, Switzerland; 9 Neuroscience Center Zurich, University and ETH Zurich, Zurich, Switzerland

**Keywords:** Fluorescence imaging, Fluorescent proteins, Synaptic transmission

## Abstract

Orexins (also called hypocretins) are hypothalamic neuropeptides that carry out essential functions in the central nervous system; however, little is known about their release and range of action in vivo owing to the limited resolution of current detection technologies. Here we developed a genetically encoded orexin sensor (OxLight1) based on the engineering of circularly permutated green fluorescent protein into the human type-2 orexin receptor. In mice OxLight1 detects optogenetically evoked release of endogenous orexins in vivo with high sensitivity. Photometry recordings of OxLight1 in mice show rapid orexin release associated with spontaneous running behavior, acute stress and sleep-to-wake transitions in different brain areas. Moreover, two-photon imaging of OxLight1 reveals orexin release in layer 2/3 of the mouse somatosensory cortex during emergence from anesthesia. Thus, OxLight1 enables sensitive and direct optical detection of orexin neuropeptides with high spatiotemporal resolution in living animals.

## Abstract (web summary)

OxLight1 is a genetically encoded sensor for the orexin neuropeptides. It has been applied in fiber photometry recordings and two-photon imaging in mice during a variety of behaviors.

## Main

Orexins (also known as hypocretins) are two neuropeptides with conserved structures and functions across mammals^1,2^; they are necessary for stable wakefulness and sleep–wake cycles, as illustrated by the cataplexy and sleep fragmentation displayed by orexin or orexin type-2 receptor or orexin cell knockout mice^3–5^, orexin type-2 receptor-deficient dogs^6^ and orexin-deficient humans^7–9^.

Both endogenous orexins (orexin-A and orexin-B) are produced by a subpopulation of neurons exclusively located in the lateral hypothalamus (LH)^10^. Axonal projections from these cells extend throughout the central nervous system to both the brain^11^ and spinal cord^12^, illustrating the anatomical basis for the involvement of orexin signaling in a broad range of neural functions^10,13^. Ongoing studies are revealing notable roles for orexin signals in regulating arousal and emotional states (including human panic attacks^14^) as well as risk-taking, reward perception and decision-making^15–17^. Furthermore, orexin cell activity in vivo is rapidly modulated by diverse external cues^16,18,19^ and slowly modulated by internal body state via nutrient-sensing^20,21^, suggesting that the orexin system may couple internal and external sensations to brain states and actions.

In spite of current knowledge, fundamental aspects of the orexin system such as the mechanisms controlling extracellular orexin degradation in the brain and the natural dynamics of orexins across behaviors, brain areas and timescales, remain difficult to study^17,22^. This limitation is due to a lack of tools for the direct detection of these neuropeptides with high sensitivity, molecular specificity and spatiotemporal resolution. Recently developed genetically encoded sensors derived from the engineering of a circularly permutated green fluorescent protein (cpGFP) into G protein-coupled receptors (GPCRs) started shedding light on the release of neurotransmitters^23–28^ in the brain and also offer an attractive solution to elucidate neuropeptide release. Using this approach we developed a genetically encoded neuropeptide sensor (OxLight1) that accurately reports the dynamics of endogenous orexins in the mouse brain under both one-photon and two-photon illumination.

## Results

### Development of a genetically encoded orexin sensor

**Fig. 1:**
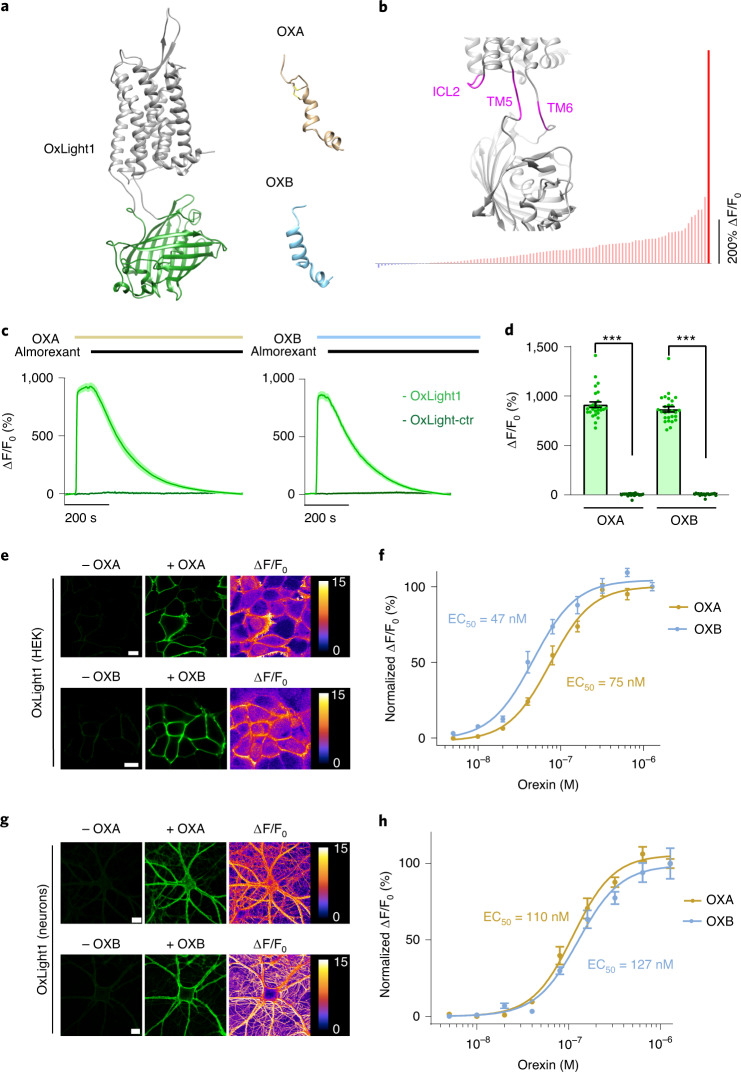
Development of the orexin sensor. **a**, Structural model of the OxLight1 sensor shown next its natural endogenous peptide ligands orexin-A (OXA; PDB, 1WSO)^42^ and orexin-B (OXB; PDB,1CQ0)^43^. **b**, Summary of the complete set of screened mutations and deletions performed in this study for a total of 101 variants. Snapshot of screening efforts, with mutated sensor regions highlighted in magenta in the structural model of OxLight1 (inset). Pink bars indicate variants with a positive response; blue bars indicate variants with a negative response. The response of OxLight1 is shown as a red bar (*n* = 5 cells for each variant). **c**, Fluorescence fold-change (Δ*F* / *F*_0_) for OxLight1 (green trace) or OxLight-ctr (gray trace) in HEK293T cells after addition of OXA or OXB each competing with almorexant. Ligand application is indicated by colored bars (all applied at 10 μM). **d**, Quantification of maximal Δ*F* / *F*_0_ from **c** for OxLight1 and OxLight-ctr. *n* = 3 independent experiments with *n* = 28, 20, 27 and 18 cells for OXA-OxLight1, OXA-OxLight-ctr, OXB-OxLight1 and OXB-OxLight-ctr, respectively. ***P < 0.0001. *P* = 2.070 × 10^−23^ and 1.242 × 10^−22^ for the response to either OXA or OXB of OxLight-ctr compared to OxLight1, respectively (two-tailed Student’s *t*-test with Welch’s correction). **e**, Representative images of OxLight1 expression in HEK293T cells and the sensor’s fluorescence intensity before (left) or after (center) 10 μM OXA (top) or OXB (bottom) application and corresponding pixel-wise Δ*F* / *F*_0_. Scale bars, 10 μm. **f**, Normalized dose–response curves (fitted with a four-parameter equation) to OXA or OXB in OxLight1-expressing HEK293T cells. *n* = 3 independent experiments with ≥18 cells each. **g**, Representative images of sensor expression in primary hippocampal neurons and the sensor’s fluorescence intensity before (left) or and after (center) 10 μM OXA (top) or OXB (bottom) application and corresponding pixel-wise Δ*F* / *F*_0_. Scale bars, 10 μm. **h**, Normalized dose–response curves (fitted as in **f**) to OXA or OXB in primary hippocampal neurons expressing OxLight1. *n* = 4 independent experiments with ≥10 neurons each. All data are shown as mean ± s.e.m. All experiments were repeated at least three times with similar results. Source data

**Extended Data Fig. 1:**
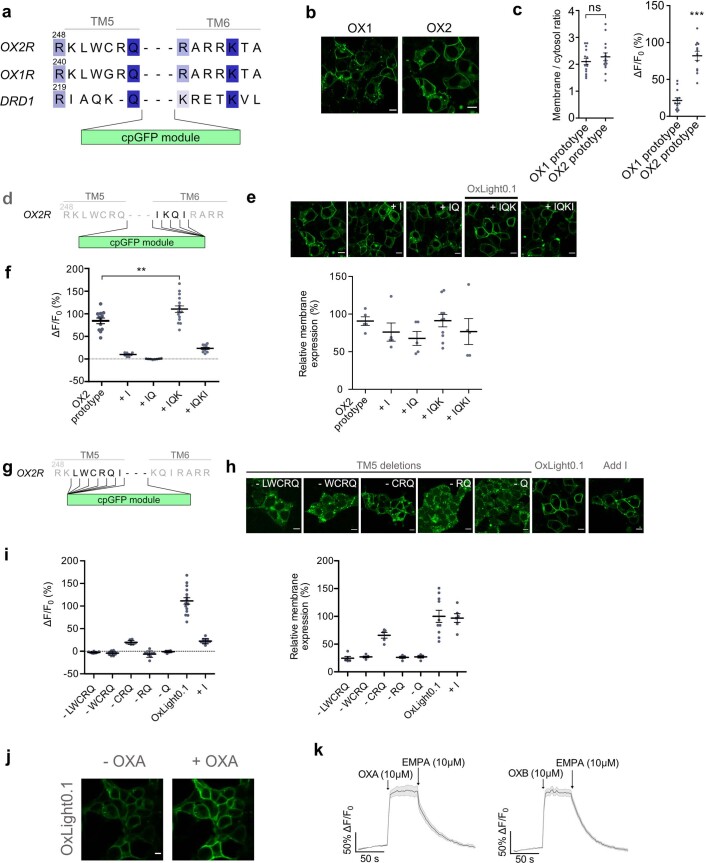
Optimization of cpGFP insertion site into OX2R. **a**, Sequence alignment of transmembrane domains (TM) 5 and 6 from OX2R, OX1R and DRD1 indicating the insertion site for the cpGFP module from dLight1. Color code is based on percent identity between orexin receptors and DRD1. **b**, Representative images showing membrane expression profile of sensor prototypes based on either the OX1 or OX2 receptor in HEK293T cells. **c**, Membrane expression and fluorescence response to 10 µM OXA of the OX1 and OX2 sensor prototypes shown in **a-b**. p = 0.3510, for the membrane expression and p = 1.109 × 10^−7^ for the fluorescence response. n = 15 cells from 3 independent experiments. **d-i**, cpGFP Insertion point optimization on the TM6 and TM5 of OX2R. The variant showing best membrane expression and response to 10 µM OXA was called OxLight0.1. **d**, Schematic representation of TM6 insertion site optimization. **e**, Representative images of TM6 insertion sites variants. **f**, Quantification of fluorescence responses to 10 µM OXA and surface expression from variants shown in **e**. p = 0.0047 for OX2 prototype response compared to OxLight0.1. n = 12 and 16 cells for OX2 prototype and OxLight0.1 or ≥ 5 cells for all other variants. **g**, Schematic representation of TM5 insertion site optimization. **h**, Representative images of TM5 insertion sites variants. **i**, Quantification of responses to 10 µM OXA and surface expression from variants shown in **h** for all variants. **j-k**, In vitro characterization of OxLight0.1 in HEK293T cells. **j**, Representative images of OxLight0.1 pre and post addition of 10 µM OXA. **k**, Response of OxLight0.1 to sequential addition of OXA or OXB followed by dual orexin receptor antagonist EMPA. n = 26 and 31 cells for OXA + EMPA and OXB + EMPA, respectively. All data are shown as mean ± s.e.m. All scale bars, 10 µm. All statistical tests performed using two-tailed student’s t-test with Welch’s correction. Source data

**Extended Data Fig. 2:**
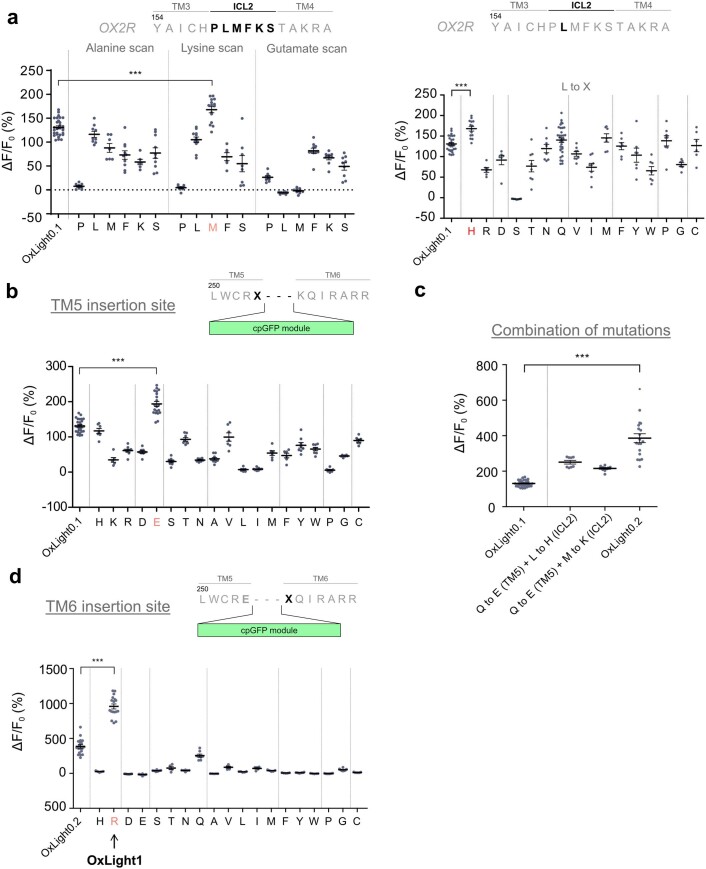
Optimization of OxLight0.1 dynamic range. **a**, Fluorescence responses to 10 µM OXA of intracellular loop 2 (ICL2) variants of OxLight0.1. Left, every amino acid from the ICL2 was individually mutated to an alanine, lysine and glutamate. Right, site-saturated mutagenesis was performed on residue L160 which corresponds to F129 in dLight1. From this screening we selected two mutations (M161K and L160H) each of whom showed significantly higher responses compared to OxLight0.1 (n = 29 cells) (∆F/F_0_ = 153 ± 8%; p = 3.704 × 10^−5^ (n = 15 cells); ∆F/F_0_ = 168 ± 6%; p = 9.954 × 10^−6^ (n = 14 cells)), respectively). **b**, Fluorescence responses of OxLight0.1 TM5 variants generated as shown in the inset. The Q254E mutant showed a significantly higher response compared to OxLight0.1 (∆F/F_0_ = 194 ± 7%; p = 1.349 × 10^−8^) n = 28 and 20 cells from 3 independent experiments for OxLight0.1 and OxLight0.1-Q to E (TM5) respectively. **c**, Combination of beneficial mutations from both the ICL2 and TM5 screenings. The triple mutant containing M161K, L160H and Q254E had a greatly improved response: ∆F/F_0_ = 386 ± 25%; p = 4.843 × 10^−9^ (n = 19 cells) and was called OxLight0.2. **d**, Fluorescence responses of OxLight0.2 TM6 variants generated as shown in the inset. The K294R mutant showed a significantly higher response compared to OxLight0.2 (∆F/F_0_ = 915 ± 14%; p = 1.075 × 10^−13^ (n = 17 cells) and was called OxLight1. n.s., not significant. All data are shown as mean ± SEM with n ≥ 5 cells. All statistical tests have been performed using a Two-Tailed Student’s t-test with Welch’s correction with data resulting from three independent experiments. *** p < 0.0001. Source data

**Extended Data Fig. 3:**
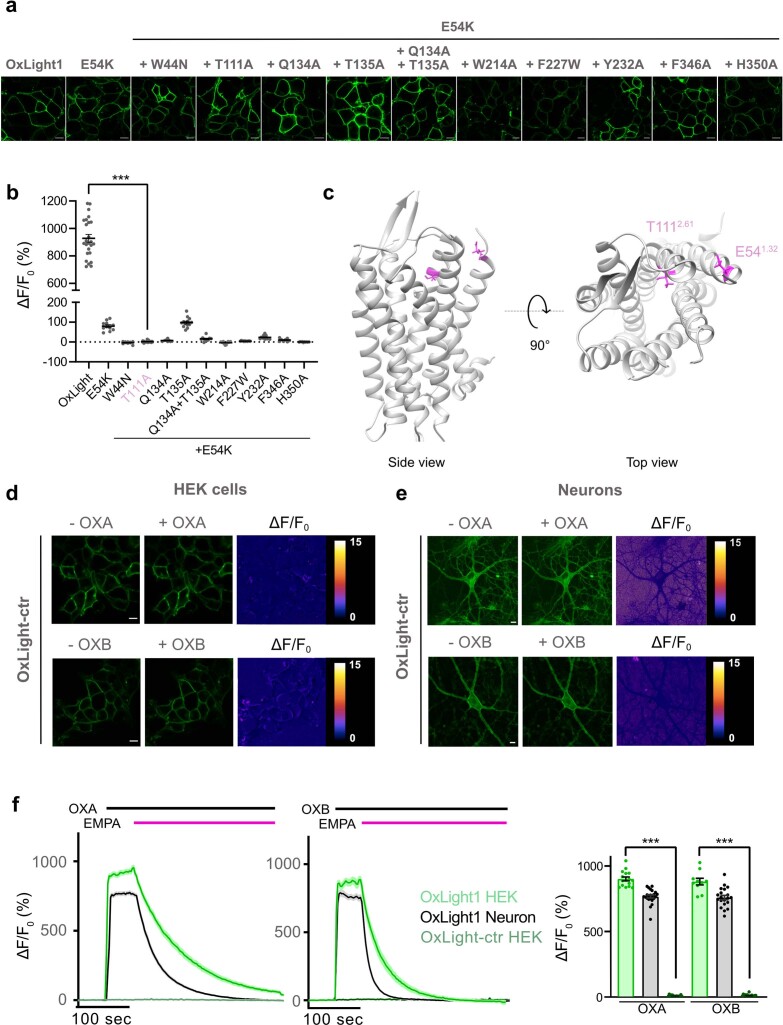
Development and characterization of a control sensor. **a**, Representative images of HEK cells expressing different mutants of OxLight1, indicated by absolute residue numbering from the N-terminus of OX2R. **b**, Quantification of maximal ∆F/F_0_ in response to bath-applied orexin-A and orexin-B (both at 10 µM) for all mutants in **a**. The OxLight1 E54K + T111A mutant has a significantly decreased response compared to OxLight1: p = 2.174 × 10^−21^ with n = 32 and 25 cells, respectively. **c**, The two OxLight1 sites selected for the control sensor are highlighted in magenta in the structural model of OxLight1. Residue sidechains are shown for the two amino acid positions E54^1.32^ and T111^2.61^. **d**, Representative images of OxLight-ctr expression in HEK cells and ∆F/F_0_ after addition of OXA or OXB. **e**, Identical to **d**, but in primary cultured neurons. **f**, Left, membrane ∆F/F_0_ in OxLight1 or OxLight-ctr expressing HEK cells from time lapse imaging experiments. Bars indicate bath application of orexins (black, both at 10 µM) followed by the OX2R antagonist EMPA (magenta, 10 µM). Right, quantification of maximal ∆F/F_0_ from time lapse imaging experiments shown on left. n = 16 and 21, cells for OxLight1 OXA HEK compared to OxLight-ctr OXA HEK (p = 1.640 × 10^−19^). n = 10 and 22 cells for OxLight1 OXB HEK compared to OxLight-ctr OXB HEK (p = 5.926 × 10^−11^), Data are shown as mean ± SEM and all statistical analysis performed using Two tailed student’s t-test with Welch’s correction from 3 independent experiments. *** p < 0.0001. All scale bars, 10 µm. Source data

To develop a genetically encoded orexin sensor we inserted a cpGFP module from the dopamine sensor dLight1 (ref. ^23^) into each of the two human orexin receptors (OX1R and OX2R) at a site selected on the basis of sequence alignment with the dopamine receptor D1 (Extended Data Fig. 1a and Supplementary Note). Once expressed in HEK cells, the prototype based on OX2R showed better membrane expression and fluorescent response to orexin-A (Extended Data Fig. 1b,c), compared to the OX1R prototype. We chose the OX2R-based design as a starting point for further development. We systematically screened the amino acid sequence adjacent to cpGFP by deleting or adding residues from the transmembrane domains 5 (TM5) and 6 (TM6) of the receptor (Extended Data Fig. 1d–i). This screening step led to a sensor variant with good membrane expression and fluorescent response to orexin-A (Δ*F* / *F*_0_ = 131 ± 3%, mean ± s.e.m.; Extended Data Fig. 1j,k). We then focused on improving the dynamic range of this variant by scanning alanine, glutamate or lysine mutations over the entire second intracellular loop (ICL2) of the receptor and identified two beneficial mutations (L16034^.51^H and M16134^.52^K; Extended Data Fig. 2a). Next, we performed site-saturated mutagenesis of both receptor residues adjoined to cpGFP. We identified two additional mutations (Q254^5.69^E and K294^6.25^R) that further enhanced the dynamic range of the sensor (Δ*F* / *F*_0_ = 906 ± 28% to orexin-A; Δ*F* / *F*_0_ = 859 ± 27% to orexin-B, mean ± s.e.m.; Fig. 1a–c and Extended Data Fig. 2b–d), which we named OxLight1 (Fig. 1a,b). We also developed a control sensor variant (OxLight-ctr) where orexin binding is abolished by the introduction of two point mutations (E54^1.32^K and T111^2.61^A; Extended Data Fig. 3a–c). While OxLight-ctr localizes well to the cell membrane (Extended Data Fig. 3a), it does not respond to orexins in HEK cells or in neurons (Fig. 1c,d and Extended Data Fig. 3b–f).

### In vitro characterization of OxLight1

**Extended Data Fig. 4:**
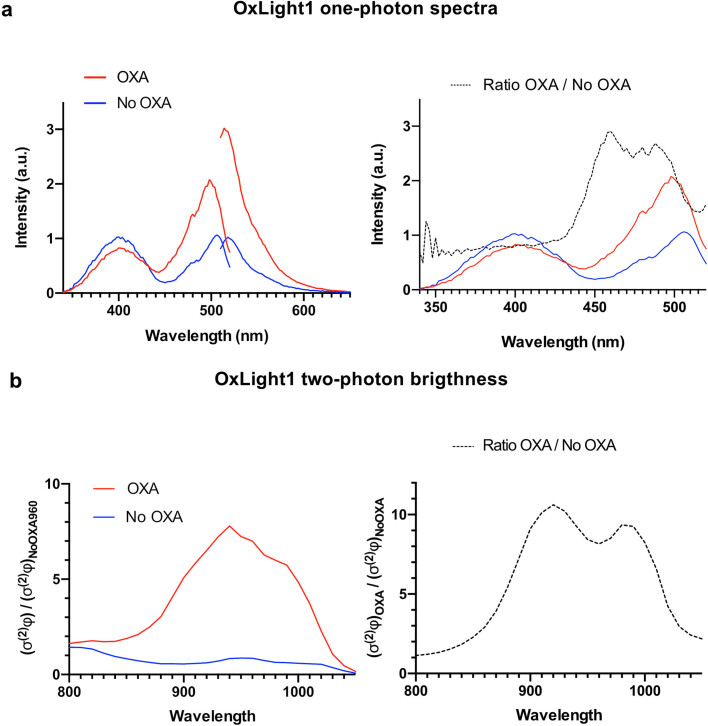
Spectral properties of OxLight1. **a**, Left, One-photon fluorescence excitation (λ emission = 560 nm) and emission (λ excitation = 470 nm) spectra acquired from OxLight1-expressing HEK cells in the absence (No OXA) or presence (OXA) of orexin-A (OXA, 10 µM). Each trace is the average of 3 independent experiments (a.u. = arbitrary units). Right, ratio of fluorescence excitation spectra shown on left. **b**, Left, relative two photon brightness of OxLight1 expressed in HEK cells grown adherent on a glass coverslip. Each trace is the average of 3 independent experiments. Right, ratio of traces shown on left. In both **a** and **b** fluorescence excitation and emission were normalized to the respective maximal value in the absence of OXA. Source data

**Extended Data Fig. 5:**
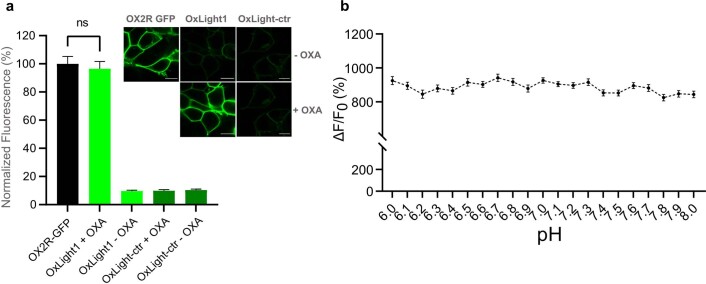
Brightness and p.H. sensitivity of OxLight1. **a**, OxLight1 and OxLight-ctr brightness assessment in the presence or absence of 10 µM OXA compared to OX2R with C-terminally tagged GFP in HEK293T cells. All the data were normalized to OX2R-GFP mean fluorescence intensity. Representative images for each condition shown on top right, scale bars, 10 µm. p = 0,6367 for OxLight1+OXA compared to OX2R-GFP, two-tailed student’s t test with Welch’s correction. n = 104 cells from 3 different experiments for each condition. **b**, Comparison of OxLight1 fluorescence response to an equimolar mix of OXA and OXB (5 µM) (black) in PBS solutions at different pH levels. n = 60; 51; 53; 62; 63; 69; 71; 66; 57; 55; 69; 73; 57; 59; 52; 61; 53; 58; 51; 46; 51 cells for each dataset from p.H. 6 to 8, respectively. Each dataset was obtained from 3 independent experiments. All data are shown as mean ± SEM. Source data

Spectral characterization of OxLight1 in HEK cells revealed that maximal signal change occurs between 460 nm and 490 nm under one-photon illumination and at 920 nm under two-photon illumination (Extended Data Fig. 4a,b). Notably, confocal imaging of sensor-expressing cells revealed that the brightness of OxLight1 in the orexin-bound state was indistinguishable from that of a GFP-tagged OX2R (Extended Data Fig. 5a). To evaluate the affinity of OxLight1 for its endogenous ligands orexin-A and orexin-B, we performed titrations of these peptides on sensor-expressing HEK cells and primary hippocampal neuronal cultures. The apparent affinity for orexin-B was in the nanomolar range (half-maximum effective concentration (EC_50_) = 47 ± 5 nM in HEK cells; EC_50_ = 110 ± 10 nM in neurons; mean ± s.e.m.), similar to that for orexin-A (EC_50_ = 75 ± 6 nM in HEK cells; EC_50_ = 127 ± 13 nM in neurons; mean ± s.e.m.; Fig. 1e–h), in agreement with previously reported affinity values for the wild-type OX2R^2,29,30^. Thus, OxLight1 senses both orexin neuropeptides with high sensitivity within the expected physiological range^15,31^.

**Fig. 2:**
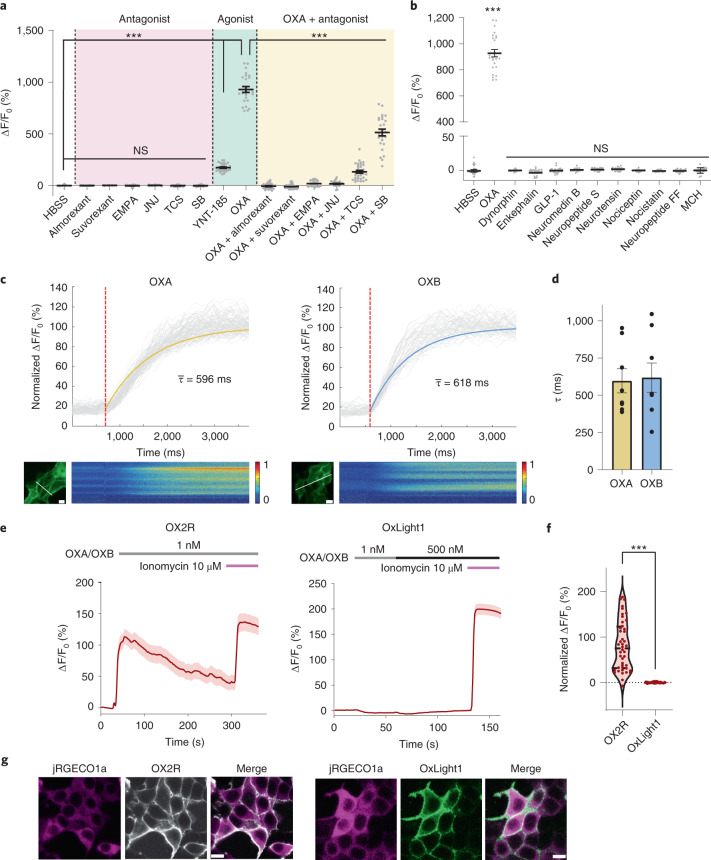
In vitro sensor characterization. **a**, Maximal Δ*F* / *F*_0_ responses in OxLight1-expressing HEK293T to addition of different drugs or drug combinations. Agonists (OXA or YNT-185 alone), OXA + antagonists (EMPA, JNJ10397049 (JNJ), TCS OX2 29 (TCS), SB334867 (SB)) and antagonists alone were applied in bolus at 10 μM final concentration. *P* values were as follows: almorexant, 0.9650 (*n* = 25 cells); suvorexant, 0.6452 (*n* = 37 cells); EMPA, 0.3013 (*n* = 28 cells); JNJ, 0.4359 (*n* = 34 cells); TCS, 0.3327 (*n* = 24 cells); SB, 0.2423 (*n* = 31 cells); YNT-185, 2.154 × 10^−24^ (*n* = 33 cells); OXA, 1.918 × 10^−21^ (*n* = 25 cells); OXA + almorexant, 3.673 × 10^−22^ (*n* = 28 cells); OXA + suvorexant, 4.286 × 10^−22^ (*n* = 18 cells); OXA + EMPA, 3.260 × 10^−22^ (*n* = 23 cells); OXA + JNJ, 6.947 × 10^−22^ (*n* = 29 cells); OXA + TCS, 6.866 × 10^−24^ (*n* = 32 cells); and OXA + SB, 1.309 × 10^−12^ (*n* = 25 cells); all antagonists and agonists were compared to Hank’s balanced salt solution (HBSS) (*n* = 38 cells) and all OXA + antagonists were compared to OXA using two-tailed Student’s *t*-test with Welch’s correction. All experiments were repeated three times with similar results. **b**, Fluorescence responses of OxLight1 to a high concentration (10 µM) of different neuropeptides. MCH, melanin-concentrating hormone; GLP-1, glucagon-like peptide 1. All experiments were repeated three times with similar results. NS, not significant. *P* values were as follows: OXA, 1.918 × 10^−21^ (*n* = 25 cells); dynorphin, 0.6434 (*n* = 30 cells); enkephalin, 0.0753 (*n* = 29 cells); GLP-1, 0.7942 (*n* = 30 cells); neuromedin B, 0.3163 (*n* = 30 cells); neuropeptide S, 0.1407 (*n* = 30 cells); neurotensin, 0.0690 (*n* = 30 cells); nociceptin, 0.6375 (*n* = 30 cells); nocistatin, 0.9033 (*n* = 30 cells); neuropeptide FF, 0.9996 (*n* = 30 cells); and MCH, 0.6613 (*n* = 24 cells); all peptides were compared to HBSS control (*n* = 38 cells) using two-tailed Student’s *t*-test with Welch’s correction. **c**, Time plots of Δ*F* / *F*_0_ for OxLight1 pixels from a representative line-scan trial. Red fluorescent dye signal onset is indicated by a dashed red line. OxLight1 responses to application of either orexin (10 µM) were fitted with a mono-exponential function. Curve fits are shown in yellow (OXA) and blue (OXB). Respective cells and line profiles used are shown directly underneath the time plots as well as the average time constant ($\bar \tau$) for OXA and OXB. Scale bars, 10 µm. **d**, Quantification of time constants from all curve fits. *n* = 8 dishes with ≥4 cells for each peptide. **e**, Characterization of OxLight1 coupling to intracellular calcium signaling. Intracellular calcium dynamics were measured in HEK293T expressing either OX2R or OxLight1 by monitoring the fluorescence of a coexpressed red calcium sensor (jRGECO1a). Δ*F* / *F*_0_ responses of jRGECO1a were recorded at baseline, after addition of 1 nM orexins for OX2R and 1 nM followed by 500 nM orexins for OxLight1. In both cases orexins were applied as an equimolar mix of OXA and OXB. Ligand addition is indicated by colored bars. All experiments were repeated three times with similar results, *n* = 44 and 45 cells for OX2R and OxLight1. **f**, Quantification of responses from **e**. Signals are normalized to the maximum response from the same cells after addition of 10 µM ionomycin. Individual data points represent the maximum jRGECO1a Δ*F* / *F*_0_ response of each cell after addition of 1 nM orexins. Violin plot represents the kernel density estimate of the probability density function for each sample. *n* = 3 dishes with ≥10 cells for each condition. *P* = 1.318 × 10^−12^ using two-tailed Student’s *t*-test with Welch’s correction. **g**, Representative images of cells used in **e**,**f**. OX2R was visualized with an Alexa-647-conjugated anti-FLAG antibody. Scale bars, 10 µm. All data are shown as mean ± s.e.m. ***P < 0.0001. Source data

Like other GPCR-based sensors^23–26^, OxLight1 inherited the pharmacological profile of its parent receptor. Individual application of various OX2R antagonists did not alter OxLight1 fluorescence, whereas application of a nonpeptide agonist (YNT-185, 10 µM) led to a significant but submaximal fluorescence response (*P* = 2.154 × 10^−24^, two-tailed Student’s *t*-test). The antagonist compounds tested were able to reduce OxLight1 response to orexins (Figs. 1c and 2a and Extended Data Fig. 3f).

To confirm that OxLight1 maintains high molecular specificity, we tested a panel of ten different neuropeptides endogenously expressed in the brain. As expected, we did not observe a response of OxLight1 to any of them (Fig. 2b). Particularly relevant is the lack of response to dynorphin, which is co-released by orexin neurons^32^. We then measured the activation kinetics of OxLight1 using high-speed line-scan imaging during application of either orexin-A or orexin-B. Fitting sensor signals with a mono-exponential function revealed sub-second activation time constants in response to either orexin peptide ($\bar \tau$ orexin-A = 596 ± 81 ms; $\bar \tau$ orexin-B = 618 ± 98 ms; mean ± s.e.m.; Fig. 2c,d). To evaluate whether OxLight1 is sensitive to extracellular pH, we measured its response in buffer solutions of set pH values. The response was only minimally affected and remained above 800% throughout the tested range (pH = 6–8) (Extended Data Fig. 5b).

**Extended Data Fig. 6:**
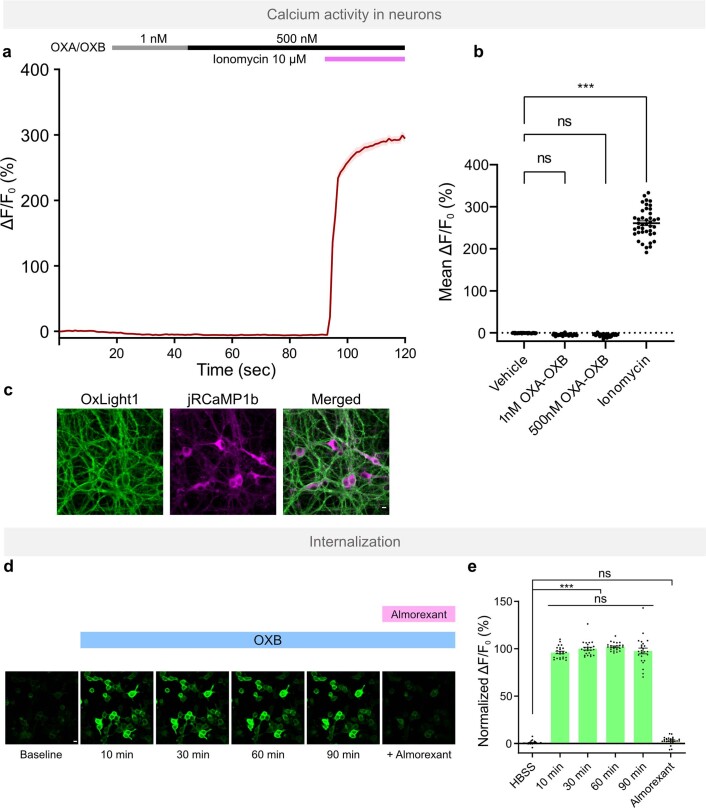
Additional characterization of OxLight1. **a**, Characterization of OxLight1 coupling to intracellular calcium activity in primary rat hippocampal neurons. Intracellular calcium dynamics were measured in co-transduced neurons expressing OxLight1 and jRCaMP1b. ΔF/F_0_ responses of jRCaMP1b were recorded after addition of vehicle (ACSF + 1 µM TTX), followed by 1 nM and 500 nM equimolar mix of OXA and OXB. Ligand addition is indicated by colored bars. Maximal intracellular calcium response was elicited with 10 µM ionomycin as a positive control. n = 41 neurons from 5 different experiments. **b**, Quantification of responses from **a**. Individual data points represent the average jRCaMP1b ΔF/F_0_ response of individual neurons for each condition. One-way ANOVA (p = 1.748 × 10^−130^) followed by Bonferroni’s multiple comparison test comparing vehicle against 1 nM OXA-OXB (adjusted p = 0,9601), 500 nM OXA-OXB (adjusted p = 0,6052) and 10 µM ionomycin (p = 9.685 × 10^−118^). ns, non-significant (p > 0.016); *** p < 0.0001 **c**, Representative images of neurons used in **a-b**. Scale bar, 10 µm. **d**, Representative images of OxLight1 fluorescence at the indicated time points before and after addition of OXB (5 µM) and almorexant (10 µM). Scale bar, 10 µm. **e**, Normalized fluorescence response values for OxLight1-expressing cells as in **d**. n = 24 cells from 3 different experiments. *** p < 0.0001, n.s. not significant. Brown-Forsythe one way ANOVA test comparing 10, 30, 60 and 90 min: p = 0.1221; Brown-Forsythe one way ANOVA test followed by post hoc Dunnett’s T3 multiple comparison test to compare HBSS to 10, 30, 60, 90 min and Almorexant. p = 0.2042 between HBSS and Almorexant. All data are shown as mean ± SEM. Source data

**Extended Data Fig. 7:**
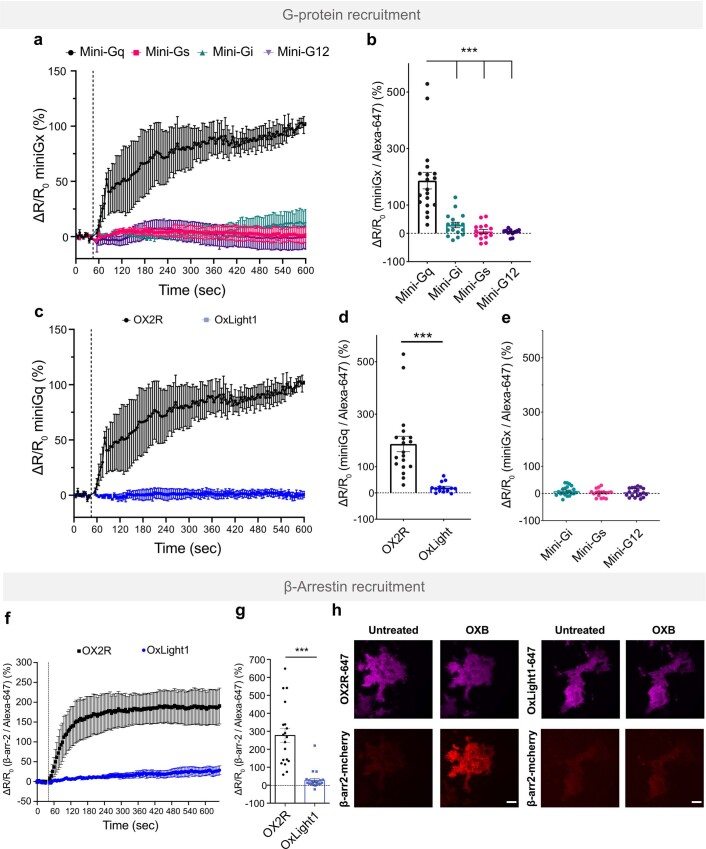
Characterization of sensor coupling to intracellular signaling partners. **a-e**, Mini-G protein recruitment to wild-type orexin type-2 receptor (OX2R) or OxLight1. **a**, Agonist-induced membrane recruitment of mRuby-tagged mini-Gq, mini-Gs, mini-Gi or mini-G12 probes (collectively mini-Gx) in OX2R-expressing HEK293 cells upon OXB addition (50 nM). TIRF time-lapse movies frame-rate was 0.2/s. Signal quantification: agonist-induced ratio-change of mRuby-fluorescence compared to baseline (∆R/R_0_). n = 3 independent experiments for each mini-G. **b**, Membrane-recruitment normalized to M1-Alexa-647 fluorescence (before vs. 5 min after OXB addition) observed for constructs tested in **a**. Mini-Gq (n = 19 cells) compared to mini-Gi (n = 17 cells, p = 4.541×10^−5^), mini-Gs (n = 15 cells, p = 7.755×10^−6^) and miniG12 (n = 11 cells, p = 6.816×10^−6^). **c**, Recruitment of mRuby-tagged-mini-Gq measured as in **a** in OX2R- or OxLight1-expressing HEK293 cells (n = 3 independent experiments). **d**, Membrane-recruitment normalized to M1-Alexa-647 fluorescence (before vs. 5 minutes after OXB treatment) observed for constructs tested in **c**. p = 2.196×10^−5^ for OxLight1 compared to OX2R. n = 19 and 14 cells for OX2R and OxLight1, respectively. **e**, Membrane-recruitment (before vs. 5 minutes after OXB treatment) of mRuby-tagged-mini-Gs, mini-Gi or mini-G12 monitored in OxLight1-expressing HEK293 cells upon OXB addition (50 nM) compared to baseline (∆R/R_0_) and normalized to M1-Alexa-647 fluorescence. n = 19, n = 21 and 17 cells (n = 3 independent experiments) for mini-G12, mini-Gi and mini-Gs respectively. **f**, Membrane-recruitment of mCherry-tagged-beta-Arrestin-2 to activated OX2R or OxLight1 upon OXB addition (50 nM), measured as in **a**. and normalized to M1-Alexa-647 fluorescence **g**, Membrane-recruitment of mCherry-tagged-beta-Arrestin-2 (before vs. 15 min after OXB addition) observed for each construct tested in **f**. p = 4.603×10^−6^ for OxLight1 compared to OX2R. n = 19 and 23 cells (n = 5 independent experiments for OX2R and OxLight1, respectively). **h**, Representative images of cells from **g** at baseline and 15 minutes after stimulation with OXB (50 nM). Scale bars, 10 µm. All data shown as mean ± SEM, two-tailed student’s t test with Welch’s correction for all statistical analyses. Source data

Next, we investigated the coupling of OxLight1 with downstream cellular signaling pathways. Stimulation of human OX2R-expressing HEK293T cells with both orexin-A and orexin-B led to robust intracellular Ca^2+^ responses (Fig. 2e–g), due to the known Gq-coupling of this receptor^2,33^. In contrast, stimulation of OxLight1 with orexins did not lead to a noticeable increase in intracellular Ca^2+^ even at high concentration (500 nM) in HEK cells or in neurons (Fig. 2e–g and Extended Data Fig. 6). To more precisely establish the G protein coupling ability of the human OX2R receptor, we monitored direct membrane recruitment of four different mini-G protein probes^34^ using total internal reflection fluorescence (TIRF) microscopy. Activation of OX2R led to selective and strong recruitment of mini-Gq, whereas little recruitment was observed for either mini-Gs, mini-Gi or mini-G12 (Extended Data Fig. 7a,b). Using this system, we then confirmed that OxLight1 activation did not elicit detectable recruitment of any of the mini-G probes tested (Extended Data Fig. 7c–e). We next monitored the recruitment of β-arrestin-2 to the cell membrane upon prolonged periods (15 min) of agonist stimulation. OX2R activation led to robust β-arrestin-2 engagement, which was not significant for OxLight1 (*P* = 4.603 × 10^−6^, two-tailed Student’s *t*-test; Extended Data Fig. 7f–h). Additionally, we verified that the sensor fluorescence response remained stable over a 1.5-h-long imaging period, after which it could be fully reversed by application of an antagonist (almorexant, 10 µM; Extended Data Fig. 6d,e). Our results indicate minimal potential for OxLight1 interference with endogenous signal transduction pathways.

### Validation of OxLight1 ex vivo and in vivo

**Fig. 3:**
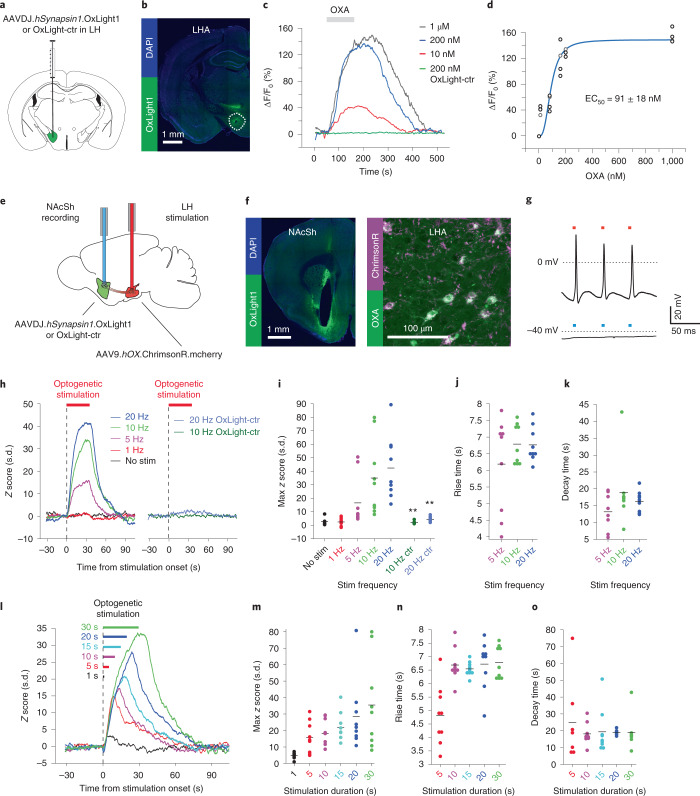
Ex vivo and in vivo validation of the sensor. **a**, Schematic drawing of AAV injections into the LH used for ex vivo validation. **b**, Representative confocal image of OxLight1 expression in LH. DAPI, 4,6-diamidino-2-phenylindole. **c**, Representative Δ*F* / *F*_0_ response traces for either OxLight1 or OxLight-ctr recorded from acute brain slices upon perfusion of the indicated OXA concentrations. **d**, Dose–response plot maximal OxLight1 Δ*F* / *F*_0_ responses in slices to different concentrations of perfused OXA. Individual data points are shown as aligned dots. Data were fitted using Hill’s equation (EC_50_ mean ± s.e.m. shown). *n* ≥ 3 slices per each concentration from four mice. **e**, Schematic drawing of viral injections and optic fiber implants in nucleus accumbens shell (NAcSh) and LH, used for in vivo photometry and optogenetic experiments. **f**, Histological verification of OxLight1 expression in NAcSh (left). Histological images showing that ChrimsonR-expression (magenta) colocalizes with OXA containing neurons of the LH (green), detected by immunostaining (right). **g**, Electrophysiological characterization of the light sensitivity of ChrimsonR-expressing orexinergic neurons. Representative recordings of *n* = 5 cells. Light stimuli were red bars, 635 nm laser; blue bar, 465 nm LED. **h**, Averaged OxLight1 fluorescence responses to increasing frequencies of optogenetic stimuli: 1, 5, 10 and 20 Hz, *n* = 3 mice, *n* = 3 randomly interleaved repeats per frequency (left). OxLight-ctr traces during 20 Hz and 10 Hz optogenetic stimulation (right). *n* = 3 mice, *n* = 3 randomly interleaved repeats per frequency. **i**, Quantification of peak fluorescence during optogenetic stimulation in **h**. Black bars indicate means. Two-sided rank-sum comparison of 20 Hz and 10 Hz for OxLight1 versus OxLight-ctr sensor was *P* = 0.0004, with no multiple comparison adjustments. **j**,**k**, Time constants of rise (**j**) and decay (**k**) in the fluorescence responses to different stimulation frequencies. **l**, Average OxLight1 fluorescence responses to optogenetic stimulus frequency of 10 Hz with train durations of 1, 5, 10, 15, 20 or 30 s, *n* = 3 mice and *n* = 3 randomly interleaved repeats per stimulation duration. Experiments were performed under isoflurane anesthesia. **m**, Quantification of peak fluorescence response during optogenetic stimulation in **l**. **n**,**o**, Time constants of rise (**n**) and decay (**o**) in the fluorescence responses to different stimulation durations. Source data

The properties of OxLight1 were further characterized in brain slices and in vivo (Fig. 3). We injected adeno-associated viruses (AAVs) carrying either OxLight1 or OxLight-ctr driven by the human synapsin promoter in the mouse LH (AAVDJ.*hSynapsin1*.OxLight1/OxLight-ctr; Fig. 3a). At 2–3 weeks after injection we prepared acute brain slices and observed sensor fluorescence under epifluorescence illumination (Fig. 3b). Perfusion of increasing concentrations of orexin-A onto the slices led to dose-dependent increases in the fluorescence of OxLight1, revealing an in situ affinity similar to the one obtained in cultured neurons (EC_50_ = 91 ± 18 nM, mean ± s.e.m.; Fig. 3c) and a maximal Δ*F* / *F*_0_ response of 155% (Fig. 3c,d). By contrast, we did not observe a response for the control sensor at the highest concentration of orexin-A tested (Fig. 3c).

To achieve precise optical control over the release of endogenous orexins, we transduced mouse LH neurons with an AAV carrying the red-shifted excitatory opsin ChrimsonR under the control of a synthetic human orexin promoter (AAV9.*hOX*.ChrimsonR; Fig. 3e)^35^. Histological characterization verified that this vector led to transgene expression in orexinergic neurons (Fig. 3f and Supplementary Fig. 1a), thus permitting optical control of orexin release. We established in slices the optical excitability of ChrimsonR-expressing orexinergic neurons by recording their membrane potential during application of blue (465 nm) or red (635 nm) light pulses. Red light reliably induced action potentials in orexinergic neurons, whereas blue light stimulation did not (Fig. 3g).

Projections from orexinergic neurons abundantly innervate the nucleus accumbens shell (NAcSh)^11^. We thus combined optogenetic stimulation of orexinergic neurons with photometry recordings of OxLight1 (or OxLight-ctr) in the NAcSh to precisely establish the relationship between orexinergic neuron activity and orexin release in vivo (Fig. 3e,f and Supplementary Fig. 1b). Mice were anesthetized, to reduce background activity of orexin neurons. We first established accumbal orexin release in response to a 30 s train of red laser light pulses of increasing frequency. While we observed little orexin release in response to 1 Hz stimulation, we detected increasing sensor responses upon stimulation with light pulses of 5, 10 and 20 Hz, reaching a maximal *z* score of 42 s.d. in response to 20 Hz (Fig. 3h,i). To further confirm that the observed signals were due to orexin release, we verified that stimulation at either 10 or 20 Hz did not elicit responses in control OxLight-ctr-expressing animals (Fig. 3h). The rise and decay times of OxLight1 signals observed during and after 20 Hz stimulation were 6.77 s and 16.31 s, respectively (Fig. 3j,k). Next, we used a fixed frequency of optical stimulation (10 Hz) and tested trains of optical stimuli of increasing duration. We found that a 30-s train of optical stimulation led to maximal OxLight1 activation (*z* score of 35 s.d.; Fig. 3l,m), which was characterized by a rise time of 6.79 s and a decay time of 18.86 s (Fig. 3n,o). Thus OxLight1 detects optogenetically evoked release of orexins in living animals with high sensitivity and rapid kinetics.

### Monitoring orexin dynamics during natural behaviors

**Fig. 4:**
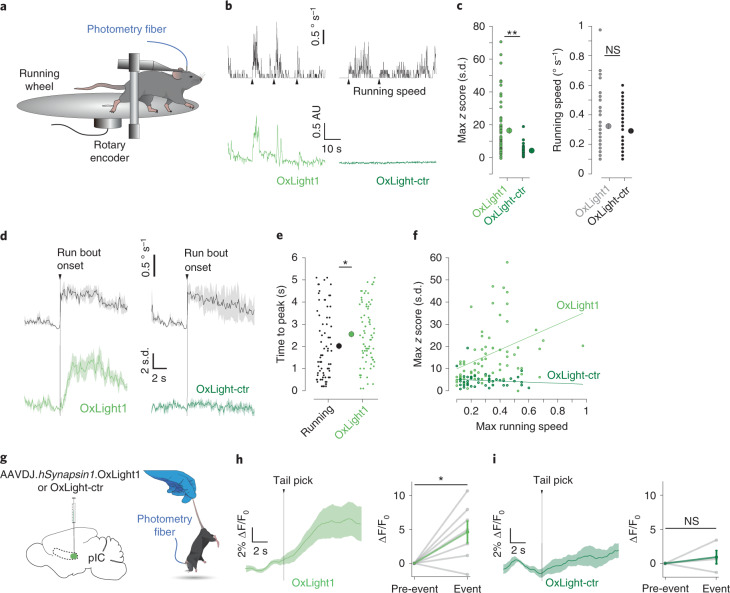
Monitoring orexin dynamics associated with natural behaviors. **a**, Schematics of experimental setup for running experiments. **b**, Example traces of OxLight1 and OxLight-ctr fluorescence during spontaneous running bouts in head-restrained mice on a running wheel. Example running bouts, onsets indicated with black arrowheads (top). Running bouts in an OxLight1 (left) and OxLight-ctr mouse (right). Running-related sensor activity in an OxLight1 (light green) and OxLight-ctr (dark green) mouse (bottom). AU, arbitrary units (raw fluorescence). **c**, Average values during onset of spontaneous running bouts. OxLight1 activity, *n* = 5 mice, n = 75 bouts (light-green circle) versus OxLight-ctr activity, *n* = 3 mice, *n* = 45 bouts (dark-green circle) (left) Two-sided rank-sum comparison of OxLight1 versus OxLight-ctr activity, *P* = 1.1537 × 10^−10^. Running speed in OxLight1 mice (gray circle) and OxLight-ctr mice (black circle) (right). Two-sided rank-sum comparison of running activity, *P* = 0.4122, with no multiple comparison adjustments (values expressed as mean ± s.e.m.). **d**, Average running-related OxLight1 activity aligned to spontaneous running bout onset. OxLight1, *n* = 5 mice, n = 75 running bouts (left); OxLight-ctr, *n* = 3 mice, n = 45 running bouts, *z*-scored traces expressed as average ± s.e.m. (right). **e**, Time to peak for run bouts versus orexin release for period from run bout onset to 5 s. Individual time to peaks for run bouts, *n* = 5 mice, n = 75 running bouts (small black circles) and average (large black circle) (left). Individual peak running times (small green circles) and average time to peak (large green circle) (right). Two-sided rank-sum comparison of time to running peak versus OxLight1 activity peak, *P* = 0.0113, values expressed as average ± s.e.m. **f**, Correlation between running peaks and OxLight1 peaks from run bout onset to 5 s., *n* = 5 mice, n = 75 bouts (light-green circles), linear regression and F-test, *R*^2^ = 0.108, *F* = 8.853, *P* = 0.0039. OxLight-ctr activity, *n* = 3 mice, *n* = 45 bouts (dark-green circles), R^2^ = 0.0125, *P* = 0.465. **g**, Schematic of injections and manipulations for tail-picking experiments. **h**, Left, ∆F/F_0_ (%) of OxLight1-injected mice (*n* = 7 mice, one episode per mouse). Light-green line represents mean response from all animals and green shading represents s.e.m. Time 0 is aligned to tail pick (vertical gray line). Mean ∆F/F_0_ (%) for the pre-event period (5 s before tail pick) and event period (10 s of tail pick) (right). Two-sided paired Student’s *t*-test (*P* = 0.027). **i**, Same as in **h** for OxLight-ctr-injected mice (*n* = 4 mice, one episode per mouse). Two-sided paired Student’s *t*-test (*P* = 0.419) (right). Source data

During wakefulness, orexin neuron activity in the LH is associated with a variety of actions and stimuli, including arousal, locomotion initiation^35^ and reward seeking^36^. Prompted by the rapid kinetics of the sensor, we tested whether OxLight1 is sensitive enough to report orexin transients associated with naturalistic behaviors. We first performed fiber photometry in mice expressing OxLight1 in NAcSh neurons. Mice were awake and head-fixed on a treadmill equipped with a rotary encoder, so that we could acquire stable photometry signals during spontaneous running (Fig. 4a). OxLight1 showed positive responses during running bout initiation that peaked approximately 0.5 s after the running activity peak (Fig. 4b–e). The amplitude of running-associated OxLight1 signals was significantly correlated with running speed (*R*^2^ = 0.108, *F* = 8.853, *P* = 0.0039, linear regression and F-test; Fig. 4f). We did not observe such transients during running in control mice expressing the mutated sensor OxLight-ctr (Fig. 4b–d).

We then tested whether orexin release could be detected in the insular cortex. Due to the known involvement of the posterior insular cortex (pIC) in the processing of aversive states^37^, we monitored orexin release during an acutely stressful event. We transduced the pIC of mice with either AAVDJ.*hSynapsin1*.OxLight1 or AAVDJ.*hSynapsin1*.OxLight-ctr and performed photometry recordings during tail-picking (Fig. 4g). After lifting the mouse by the tail we observed a noticeable and significant increase in OxLight1 fluorescence (*P* = 0.027, two-sided paired Student’s *t*-test; Fig. 4h). In contrast, control recordings using the mutated sensor OxLight-ctr showed no transients in response to the same handling protocol (Fig. 4i). Taken together these results indicate that OxLight1 faithfully reports the endogenous release of orexins in response to naturalistic behaviors.

### Tracking orexin dynamics across sleep–wake cycles

**Fig. 5:**
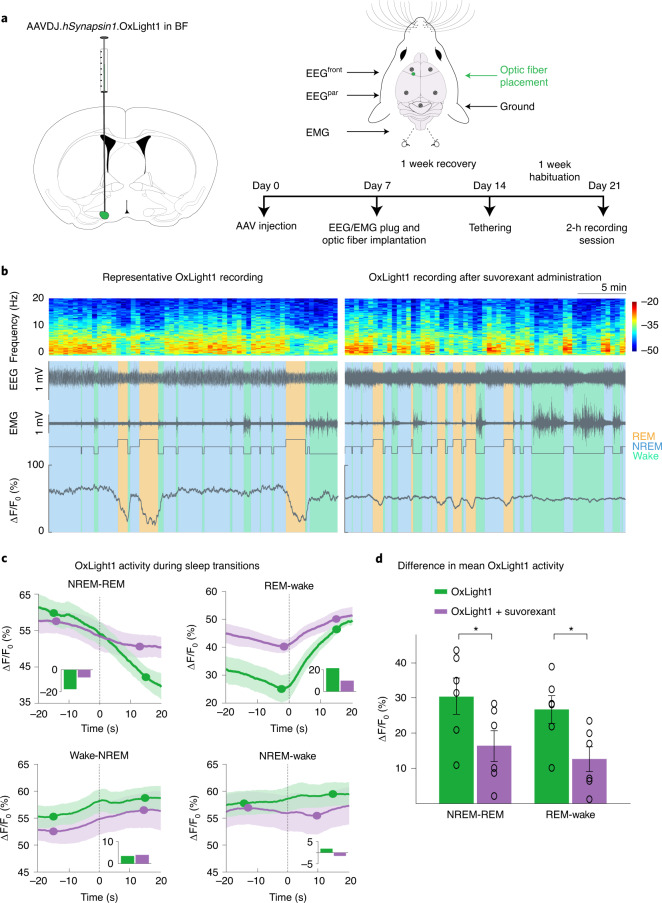
Tracking orexin dynamics across sleep–wake cycles. **a**, Schematic drawing of AAV injections in the BF and experimental setup for EEG, EMG and fiber photometry recordings during sleep–wake states. **b**, EEG spectrogram, EEG, EMG, hypnogram and Δ*F* / *F*_0_ fluorescence recordings of an OxLight1-injected animal with or without suvorexant administration (top to bottom). Representative 30-min OxLight1 recordings without pharmacological treatment (left). Representative recordings after suvorexant administration (dosage, 50 mg kg^−1^) in the same animal (right). Hypnogram color codes are wake, green; NREM, blue; and REM, orange. **c**, Changes in average Δ*F* / *F*_0_ of OxLight1-expressing neurons at the transition of the different sleep–wake states in the presence or absence of suvorexant. OxLight1 recordings performed in the absence of pharmacological treatment are shown in green, whereas recordings in the presence of suvorexant are shown in purple (*n* = 6 mice). Plotted changes in Δ*F* / *F*_0_ for each condition (treated versus untreated) represent pooled data from four animals implanted and recorded in the BF and two animals implanted and recorded in the LH. Transition from NREM to REM and from REM to wake (top). Transition from wake to NREM and from NREM to wake (bottom). Bar plots represent the difference in OxLight1 activity (normalized Δ*F* / *F*_0_) between maximum and minimum of the 15-s time point before and after the state transition. **d**, Bar plots showing the difference between mean Δ*F* / *F*_0_ of the sleep–wake states in OxLight1-injected animals between the nontreated and suvorexant-treated condition (*n* = 6 mice). Difference of Δ*F* / *F*_0_ NREM-REM (two-way ANOVA with Bonferroni’s multiple comparisons test; *P* = 0.0269) (left); difference of Δ*F* / *F*_0_ REM-wake (two-way ANOVA with Bonferroni’s multiple comparisons test; *P* = 0.0277) (right). All data are shown as mean ± s.e.m. Source data

**Extended Data Fig. 8:**
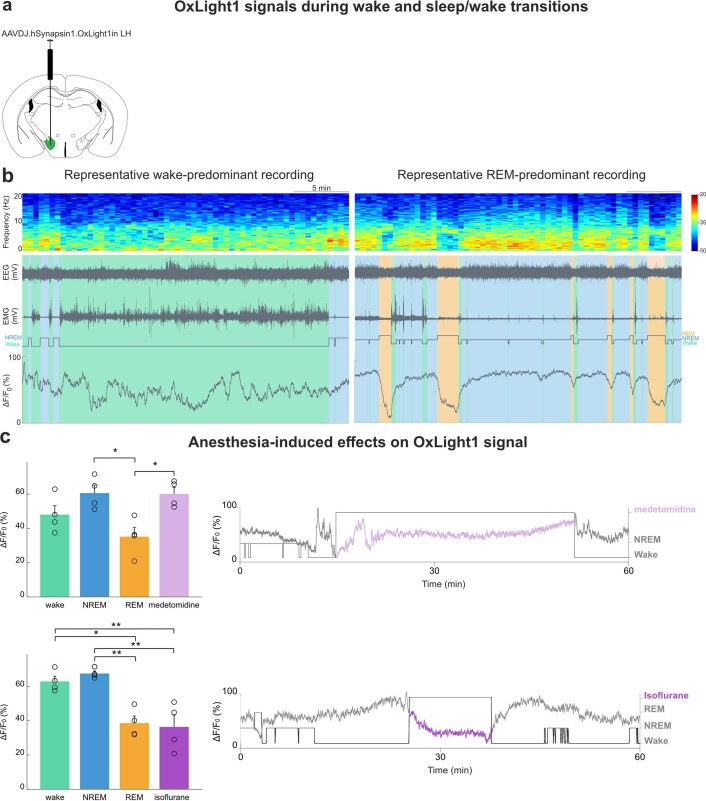
Dynamic orexin fluctuations during brain states. **a**, Schematic drawing of AAV injections in the LH and experimental setup for recording EEG, EMG and fiber photometry during sleep–wake states. **b**, Representative recordings showing (top to bottom) EEG spectrogram, EEG, EMG, hypnogram, and ΔF/F_0_ fluorescence trace of an OxLight1-injected animal. Left, example of a wake-predominant 30-minute recording, and right, example of a REM-predominant 30-minute recording. Both traces belong to the same OxLight1-injected animal. Hypnogram color-code: wake = green, NREM = blue, REM = orange. **c**, Medetomidine and isoflurane-induced modulation of OxLight1 signal in OxLight1-injected mice. Left, bar plots graph showing mean ΔF/F ± SEM of the sleep–wake and anesthetic-dependent states (medetomidine top left, isoflurane bottom left). Statistical analysis was performed using one-way ANOVA with Bonferroni’s multiple comparisons test n = 4 mice, p = 0.0191 for NREM vs REM (Medetomidine), p = 0.0216 for REM vs Medetomidine, p = 0.0118 for Wake vs REM, p = 0.0062 for Wake vs Isofuorane, p = 0.0032 for NREM vs REM (isoflurane), p = 0.0017 for REM vs Isofluorane. Right, representative hypnogram and trace of OxLight1 fluorescence in one-hour recording during medetomidine injection (top right) and isoflurane exposure (bottom right). Time spent under isoflurane-induced anesthesia: 12 minutes, time spent in medetomidine-induced anesthesia: 30 minutes. Source data

Orexin neurons and orexin receptors are important for the regulation of sleep and wakefulness^22,38^. This motivated us to test whether, using our sensor, we could obtain high-resolution information on orexin release across sleep–wake cycles. We performed photometry recordings of OxLight1 in two distinct deep brain regions during polysomnographic recordings. Mice received injections of AAVDJ.*hSynapsin1*.OxLight1 and chronic implantation of an optic fiber for photometry recordings over the basal forebrain (BF) or the LH, as well as the implantation of electrodes for simultaneous electroencephalographic (EEG) and electromyographic (EMG) recordings (Fig. 5a and Extended Data Fig. 8a). We found that the OxLight1 signal exhibited large fluctuations during wake and remained on average higher during wakefulness and non-REM (NREM) sleep compared to REM sleep (Extended Data Fig. 8b). At the onset of REM sleep, the signal rapidly decreased and remained low for the duration of the REM sleep episode, only to rapidly increase back during the transition from REM sleep to wake (Fig. 5b–d and Extended Data Fig. 8b). Furthermore, administration of suvorexant (50 mg kg^−1^, per os) significantly reduced the response of OxLight1 across sleep–wake states (NREM-REM, *P* = 0.0269; REM-wake, *P* = 0.0277, two-way analysis of variance (ANOVA); Fig. 5b–d). We also investigated the effects of deep anesthesia on orexin levels detected by OxLight1. While we observed a decrease in OxLight1 signal in response to isoflurane, we did not detect a change when we induced anesthesia using medetomidine (Extended Data Fig. 8c). Thus, different anesthetic agents seemed to distinctively affect the release of orexins, likely due to the engagement of different molecular targets^39^.

### Two-photon imaging of cortical orexin release

**Fig. 6:**
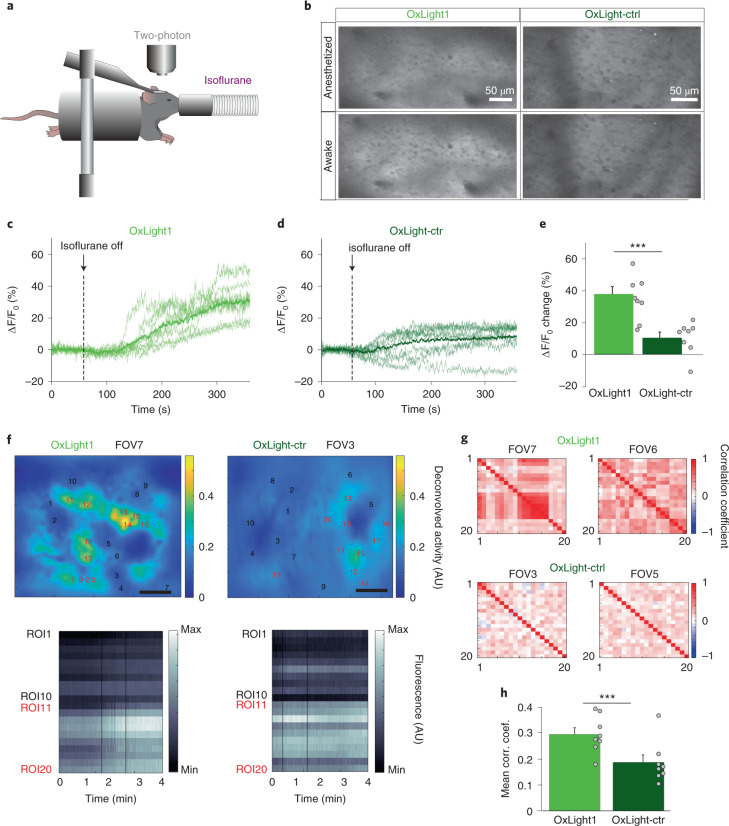
Two-photon imaging of cortical orexin dynamics during emergence from anesthesia. **a**, Experimental setup. Two-photon imaging was performed in the somatosensory cortex of mice resting inside a plastic tube. Isoflurane anesthesia was delivered for the first minute of the acquisition. **b**, Maximum intensity projections from one FOV in a mouse expressing OxLight1 in layer 2 of the somatosensory cortex (left) and one from a mouse expressing the control vector in the same region (right). Projections resulting from the first minute of imaging, during which mice received isoflurane (top). Projections obtained from the frames acquired during the last minute of imaging, when isoflurane anesthesia had been off for 5 to 7 min (bottom). Range of pixel intensities is the same for the two OxLight1 projections and for the two projections from the control FOV. **c**, Fluorescence traces representing the frame-by-frame average fluorescence from all pixels in eight FOVs from four mice expressing OxLight1. **d**, Same as in **c**, but for eight FOVs from four mice expressing the control vector. **e**, Difference between mean Δ*F* / *F*_0_ values during the first minute and the last minute of imaging in the eight FOVs shown in **c** and the eight FOVs shown in **d** (mean ± s.e.m.). Individual data are shown as gray dots and were jittered to improve visibility. One-sided unpaired Student’s *t*-test; *P* = 5.078 × 10^−4^. **f**, Average projection of one OxLight1 (top left) and one OxLight-ctr (top right) FOV after frame-by-frame binarization and deconvolution of the raw data. Black numbers indicate ROIs identified as inactive, whereas red numbers indicate active ROIs. Scale bar, 50 µm. Heat maps showing the raw fluorescence of the 20 ROIs identified in the OxLight1 (bottom left) and OxLight-ctr FOVs, during 4 min of imaging starting 1 min after anesthesia delivery was stopped. **g**, Pearson’s correlation coefficients between all ROI pairs in two example OxLight1 FOVs (top) and two OxLight-ctr FOVs. **h**, Average Pearson’s correlation coefficient for the ROI pairs in each OxLight1 FOV (*n* = 8) and in each OxLight-ctr FOV (*n* = 8). Individual data are shown as gray dots and were jittered to improve visibility. OxLight-ctr data were not normally distributed. One-sample Wilcoxon signed-rank test was used to analyze data, *P* = 0.007. Source data

**Extended Data Fig. 9:**
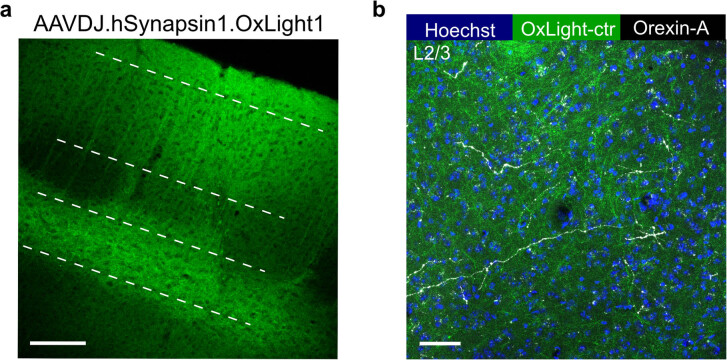
Immunohistochemical verification of orexinergic fibers in the somatosensory cortex. **a**, A coronal section of the somatosensory cortex of one mouse showing the laminar spread of OxLight1. Scale bar, 100 µm. **b**, A high-magnification image (40x) of one coronal section from a brain expressing OxLight-ctr (green) in the somatosensory cortex layer 2/3 (L2/3). The section was stained with Hoechst dye (blue) and with an anti-Orexin-A antibody (white). Scale bar, 50 µm.

**Extended Data Fig. 10:**
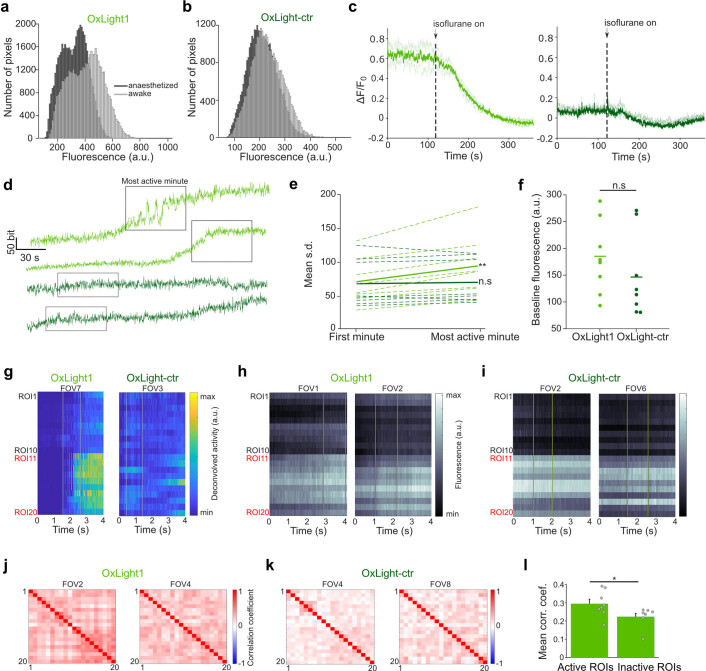
Characterization of OxLight1 and OxLight-ctr expression in somatosensory cortex. **a**, Distributions of pixel-intensities for maximum-projections shown in Fig. 6b top-left (dark grey) and bottom left (light grey) from one FOV in a mouse expressing OxLight1. **b**, Same as in **a** for OxLight-ctr, related to Fig. 6b top-right (dark grey) and bottom right (light grey). **c**, Fluorescence traces representing frame-by-frame average fluorescence from all pixels in 2 FOVs from 2 mice expressing OxLight1 (left) or OxLight-ctr (right). Anesthesia was off for the first 2 minutes of imaging. **d**, Raw-fluorescence traces for 2 example OxLight1-FOVs and 2 example OxLight-ctr-FOVs, corresponding to a time window between the 3rd and 6th minute of imaging in each FOV. Boxes highlight the most-active minute. **e**, Mean across-frames s.d. calculated during the first minute of imaging and during the most-active minute, for each OxLight1 FOV (light-green) and OxLight-ctr-FOV (dark-green). Solid lines indicate mean across FOVs. OxLight1 data are normally distributed (paired t-test; p = 0.0013), while OxLight-ctr data are not (Wilcoxon signed-rank test; p = 0.57). **f**, Median pixel intensity values during the anaesthetized period (F0) in 8 OxLight1-FOVs and 8 OxLight-ctr-FOVs. The two distributions are not significantly different (unpaired t-test; p = 0.41). **g**, Heatmaps showing the frame-by-frame deconvolved activity in 20 ROIs from one example OxLight1-FOV (left) and one example OxLight-ctr-FOV (right). Solid vertical grey lines indicate the most-active minute. FOVs are the same as in Fig. 6 f. **h**, Heatmaps showing frame-by-frame raw fluorescence of each ROI in 2 example OxLight1-FOVs. Yellow lines indicate the most-active minute. **i**, Same as in h, but for 2 example OxLight-ctr-FOVs. **j**, Pearson’s correlation coefficients between all ROI pairs in 2 example OxLight1-FOVs, during the most-active minute. **k**, Same as in j, but for two example OxLight-ctr-FOVs. **l**, Pearson’s correlation coefficients in 8 OxLight1-FOVs across all pairs of active and inactive ROIs, during most-active minute (mean + /- SEM) (Wilcoxon signed-rank test; p = 0.0207). Source data

Because OxLight1 is excitable through a two-photon absorption process (Extended Data Fig. 4b), we performed two-photon OxLight1 imaging in vivo to test whether the cortex receives dynamic orexin signals during the transition between anesthesia and wakefulness. We first expressed OxLight1 or OxLight-ctr in the superficial layers of the mouse somatosensory cortex, where we verified the presence of orexinergic axon terminals (Extended Data Fig. 9a,b). We then monitored the indicator’s fluorescence during the emergence from isoflurane anesthesia (Fig. 6a). Fluorescence values across the field of view (FOV) gradually increased in mice expressing OxLight1 as they woke up (Fig. 6b, Extended Data Fig. 10a,b and Supplementary Video 1). The gradual increase in fluorescence started about 1 min after we stopped isoflurane delivery and reached a plateau 4 min later (Fig. 6c–e). The difference between the median FOV fluorescence measured during the first and the last min of imaging was significantly higher in OxLight1-expressing mice compared to OxLight-ctr expressing mice (*P* = 5.08 × 10^−4^, one-sided unpaired Student’s *t*-test; Fig. 6d,e and Supplementary Video 2). The average fluorescence signal dropped in OxLight1 mice more than in OxLight-ctr when we started imaging in awake mice and turned isoflurane on during the acquisition (Extended Data Fig. 10c).

To investigate the dynamics underlying the overall increase in orexin levels during emergence from anesthesia, we focused on a 4-min-long period beginning when OxLight1 signal started rising and ending when the signal reached its plateau. Here we identified the 1 min carrying the highest fluorescence variability across frames for each FOV (the ‘most-active minute’; Extended Data Fig. 10d). The across-frame variability during this time window was on average significantly higher than the variability during the first minute of imaging in OxLight1 FOVs but not in OxLight-ctr FOVs (paired Student’s *t*-test for OxLight1, *P* = 0.001; Wilcoxon signed-rank test for OxLight-ctr, *P* = 0.57; Extended Data Fig. 10e). Notably, the F_0_ distributions were not significantly different between the two groups (*P* = 0.41, unpaired Student’s *t*-test; Extended Data Fig. 10f). Within each FOV, subregions of high and low OxLight1 activity were both present. The large-scale localization of these subregions remained stable when the same FOV was imaged twice (Supplementary Fig. 2a). Nonetheless, local differences also emerged, where OxLight1 activity was stronger either in the first acquisition or in the following one (Supplementary Fig. 2b). For each FOV, we identified ten regions of interest (ROIs) of high orexin activity and ten ROIs of low activity (Fig. 6f and Extended Data Fig. 10g–i). Pairwise Pearson’s correlations across ROIs during the most-active minute revealed clusters of highly correlated active ROIs in the OxLight1 FOVs but not in the OxLight-ctr FOVs (Fig. 6g and Extended Data Fig. 10j,k). Correlations were on average significantly higher between OxLight1 active ROIs compared to OxLight-ctr active ROIs (*P* = 0.007, one-sample Wilcoxon signed-rank test; Fig. 6h), despite significant variability across OxLight1 FOVs (*P* = 1.23 × 10^−10^, one-way ANOVA). Moreover, average correlations in OxLight1 FOVs during the most-active minute were significantly higher between active than inactive ROIs (*P* = 0.0207, Wilcoxon signed-rank test; Extended Data Fig. 10l). Overall, these results suggest that changes in orexin concentration within localized cortical regions occur during emergence from anesthesia and can be detected by OxLight1. Similar local dynamics were present when mice were fully awake (Supplementary Fig. 3).

## Discussion

Using an all-optical approach, we dissected the relationship between neuronal activity and neuropeptide release, a long-sought question in neurophysiology^17^. The unexpected observation that orexin levels rapidly drop and remain low during REM sleep indicates that the dynamics of orexins in vivo can exhibit relatively rapid changes, the amplitude and duration of which are presumably affected both by diffusion mechanisms and proteolytic activity in the extracellular milieu.

Our observations that orexin fluctuations occur in the somatosensory cortex upon emergence from anesthesia represent direct evidence showing that orexin neuropeptides are present in supragranular cortical layers (layer 2/3). Although OX2R messenger RNA expression has been observed in this cortical region in rodents^40^, functional studies reported sensitivity to orexins only in deep cortical layers of the S1 cortex (layer 6b)^41^. Our recordings suggested that cortical orexin transients display a certain degree of spatial organization, with localized microdomains displaying stronger and more correlated OxLight1 signals compared to neighboring areas. Thus, the combination of OxLight1 and high-resolution two-photon microscopy opens up the possibility of mapping orexinergic signaling with unprecedented spatial precision.

The spatial organization of OxLight1 signaling that we observed is compatible with differences in the concentration of orexin peptides across areas, although a contribution of differences in the indicator’s expression level cannot be completely ruled out. The same limitation applies to all cpGFP-based sensors, in which the lack of an internal stable reference signal prevents accurate normalization of the observed fluorescence patterns to the expression levels of the probe. Future sensor development efforts should explore the possibility of incorporating a secondary stable fluorescent protein in the construct. Additional areas of development include increasing the affinity and selectivity for either orexin peptide or generating red-shifted versions of the indicator. OxLight1 will pave the way for the development of neuropeptide sensors and enable a deeper understanding of neuropeptide physiology at the cellular, network and behavioral level.

## Methods

### Molecular cloning

The sequence alignments for the initial design of the sensors were performed using Clustal Omega2 (EMBL-EBI)^44^. The sequences encoding the OX1R and OX2R receptors and corresponding prototype sensors were ordered as synthetic DNA geneblocks (Integrated DNA Technologies) flanked by HindIII and NotI restriction sites for cloning into a pEGFP–N1–FLAG plasmid (Addgene, 60360). Sequences coding a hemagglutinin (HA) cleavable secretion motif and a FLAG tag were included at the 5ʹ end of the construct to facilitate sensor expression and characterization, respectively. The C-terminally GFP-tagged OX2R construct was cloned by restriction ligation of the OX2R into pEGFP–N1–FLAG using HindIII and AgeI sites. Specific sensor mutants were obtained either using circular polymerase extension cloning^45^ or site-directed mutagenesis with custom-designed primers (Thermo Fisher). PCR reactions were performed using Pfu-Ultra II Fusion High Fidelity DNA Polymerase (Agilent). All sequences were verified using Sanger sequencing (Microsynth). For cloning OxLight1 and OxLight-ctr into the viral vector, BamHI and HindIII restriction sites were added, flanking the sensor coding sequence by PCR amplification, followed by restriction cloning into pAAV–hSynapsin1–WPRE, obtained from the Viral Vector Facility (VVF) of the University of Zurich.

### Structural modeling

The structural model of the OxLight1 sensor was generated using the Molecular Operating Environment software. Structures of the GPCR-derived segment and the cpGFP-derived segment were first obtained individually by homology modeling (Protein Data Bank IDs 5WQC and 3SG7, respectively) and then fused using the amino acid linkers described in this study. Energy minimization was performed on the system to generate the final prediction.

### Cell culture, imaging and quantification

HEK293T cells (ATCC, CRL-3216) were cultured in DMEM (Thermo Fisher) supplemented with 10% FBS (Thermo Fisher) and 100 μg ml^−1^ penicillin–streptomycin mix (Thermo Fisher) and incubated at 37 °C with 5% CO_2_. Cells were transfected at 50–60% confluency in glass-bottom dishes (either individually or in 24-well plates) using the Effectene transfection kit (QIAGEN) according to manufacturer instructions and imaged 24–48 h after transfection. Primary hippocampal neurons were obtained from E18.5 rat embryos and cultured in minimum essential medium (Thermo Fisher Scientific) containing 15 mM HEPES, 15% Nu-Serum (Corning Incorporated), 0.45% glucose, 1 mM Na-pyruvate, 2 mM l-glutamine and B27 (1×, Thermo Fisher Scientific)^46^. Neurons were transduced by direct addition of either AAV.hSynapsin1.OxLight1 or AAV.hSynapsin1.OxLight-ctr in the culture medium at a 1 × 10^10^ genome copies (GC) ml^−1^ final titer. Before imaging, cells were rinsed with 1 ml of HBSS (Life Technologies) supplemented with Ca^2+^ (2 mM) and Mg^2+^ (1 mM) and then placed in a final HBSS volume of either 100 µl for each individual 1.5-cm glass-bottom dish or 500 µl for 24-well plates. For calcium imaging experiments in neurons, cells were rinsed and imaged using a 564 nm laser for the jRCaMP1b sensor in artificial cerebrospinal fluid (aCSF) solution (120 mM NaCl, 2.5 mM KCl, 1.25 mM NaH_2_PO_4_, 26 mM NaHCO_3_, 5 mM HEPES, 1 mM MgCl_2_(6H_2_O), 14.6 mM glucose and 2.5 mM CaCl_2_(2H_2_O), pH 7.4) in the presence or absence of tetrodotoxin (1 µM), where specified. Time-lapse imaging was performed at room temperature (22 °C) on an inverted Zeiss LSM 800 confocal microscope using Zeiss Zen Blue 2018 (v.2.6) software and either a ×40 oil-based (for individual dishes) or a ×20 objective (for 24-well plates). Imaging was performed using a 488 nm laser for OxLight1 and OxLight-ctr sensors, a 564 nm laser for the jRGECO1a sensor and a 647 nm laser for Alexa-647-conjugated anti-FLAG antibody. During imaging, ligands were added in bolus on the cells using a micropipette to reach the final specified concentrations of ligands over the cells. For pH titration experiments, cells were bathed for 5–10 min before imaging in PBS (Thermo Fisher) and adjusted to specified pH levels using 0.1 M HCl or 0.1 M NaOH. For quantification, except otherwise noted, ROIs were selected manually using the threshold function of Fiji (ImageJ v.1.52) to isolate the cell membrane. Sensor response (∆*F*/*F*_0_) was calculated as (*F*(*t*) − *F*_0_) / *F*_0_, with *F*(*t*) being the ROI fluorescence value at each time point (*t*) and *F*_0_ being the mean fluorescence of the ten time points immediately before ligand addition. ∆*F*/*F*_0_ values were plotted and analyzed in GraphPad Prism 9. The ∆*F*/*F*_0_ images were produced using MATLAB (R2019a) by dividing pixel-by-pixel fluorescence intensities before and after addition of orexins. A color scale was then applied to generate an RGB image.

### Spectral characterization

Both one-photon and two-photon spectral characterizations of the sensor were performed using OxLight1-transfected HEK293T cells before and after addition of orexin-A (10 µM). One-photon fluorescence excitation (λ_em_ = 560 nm) and emission (λ_exc_ = 470 nm) spectra were determined on a Tecan M200 Pro plate reader at room temperature. At 24 h after cell transfection in a six-well format (JetPEI, PolyPlus), ~1 million cells were dissociated with addition of TrypLE Express (Thermo Fisher) and thoroughly washed with PBS. Next, cells were resuspended in 300 µl PBS and aliquoted into two individual wells of a 96-well microplate with or without orexin-A (10 µM), together with two wells containing the same amount of non-transfected cells to account for autofluorescence and a single well containing PBS for subtraction of the Raman bands of the solvent. For determining two-photon brightness of the sensor, images were taken using Scanimage 5 software and a previously reported two-photon microscope^47^ equipped with a wavelength tunable fs-laser (Chameleon Discovery, Coherent) and a ×20 Zeiss W Plan objective. The imaging plane was kept constant and the average fluorescence intensity at every excitation wavelength was measured, before and after bolus addition of orexin-A (10 µM). Laser power after the objective was calibrated at every wavelength using a beam splitter and a pair of power meters (reference, Thorlabs S121C, after objective, Thorlabs S175C). During the experiment, the power at objective was kept <30 mW to avoid photobleaching and to minimize saturation effects^48^. To account for the wavelength dependence of the pulse width due to the laser specifics and the group velocity dispersion of the optics, we measured a correction curve using the ratio between the measured and reported^49^ spectral shape of Rhodamine6G (1 × 10^−5^ M in methanol). The fluorescence emission spectra of the two forms of the sensor are nearly identical, thus a calibration for the relative detection efficiency was not necessary. Since both the two-photon absorption cross-section (σ^(2)^) and the quantum yield (Φ) can change upon ligand binding^50^, we report the relative two-photon brightness, which is related to the product of these quantities and can be calculated from our data using previously reported equations^48^.

### Sensor kinetics analysis

To study sensor activation kinetics, the red fluorescent dye Antonia Red-Dextran (molecular mass 3,000 Da, Sigma-Aldrich) was diluted to equimolar amounts (10 µM) with OXA or OXB (Tocris) in HBSS and applied in bolus onto HEK293T cells transiently transfected with OxLight1. Confocal imaging for activation kinetics was performed at 37 °C, with heating provided by a stage-top incubator (Tokai Hit). Fluorescence signals were elicited by 488 nm (OxLight1) and 561 nm (red dye) illumination and recorded simultaneously at 550 Hz using line-scan mode. Lines for recordings were selected across multiple cells. Pixels within the line were categorized as belonging to either membrane or cytosol by thresholding the change in fluorescence intensity at 488 nm before and after addition of the ligand. Only membrane pixels were used for subsequent analysis. Line-scans with latencies of red fluorescence signal onset <50 ms (defined as time from baseline to 85% of maximal fluorescence change) were selected for kinetic analysis. Fluorescent intensity of the green channel across time for each pixel was low-pass filtered and fitted to a one-phase exponential association model using custom-written MATLAB scripts to calculate the time constant τ.

### Intracellular signaling characterization

To characterize coupling to intracellular Ca^2+^ signaling, HEK293T cells were transfected with either wild-type human OX2R or OxLight1, together with the red calcium sensor jRGECO1a^51^ and were imaged 24–48 h after transfection. To visualize the FLAG-tagged OX2R receptor, cells were first incubated for 5 min at 37 °C with a 1:1,000 dilution of an Alexa-647-conjugated M1 anti-FLAG antibody in HBSS with Ca^2+^ (2 mM) and then washed twice with buffer before being imaged under confocal microscopy. To visualize stimulation of intracellular Ca^2+^ signaling by signaling of the orexin receptor, we first recorded 3–5 min of baseline activity, followed by addition of an equimolar mix of orexins at indicated concentrations. As a final step, saturation of jRGECO1a was achieved by triggering extracellular calcium entry into cells upon addition of ionomycin (10 µM, Sigma-Aldrich). To sensitively and directly monitor the recruitment of intracellular signaling proteins we used TIRF microscopy-based assay. HEK293T cells were seeded on polylysine-coated glass-bottom dishes (MatTek) and transfected using Lipofectamine 2000 (Thermo Fisher) according to manufacturer’s specifications with a combination of either wild-type OX2R or OxLight1, together with either mRuby-tagged mini-G proteins (mini-Gs, mini-Gsi, mini-Gsq and mini-G12 (ref. ^34^)) or mCherry-tagged β-arrestin-2 at a 5:1 DNA ratio. After 24–48 h of transfection, cells were imaged at 37 °C using a ×100 1.49 oil CFI Apochromat TIRF objective on a Nikon TIRF microscope equipped with temperature chamber, objective heater, perfect focus system and an Andor DU897 EMCCD camera, in time-lapse mode with 5-s intervals. The laser lines used were 561 nm (for visualizing mini-G proteins or β-arrestin-2) and 647 nm (for visualizing flag-labeling of receptor or sensor). Stimulation was performed by adding 50 nM OXB by bath application. Protein relocalization (∆*R*/*R*_0_) was calculated as (*R*(*t*) − *R*_0_) / R_0_ with *R*(*t*) being the fluorescence signal from tagged mini-G protein or β-arrestin-2 at each time point (*t*) (normalized to M1-Alexa-647 signal, when specified) and *R*_0_ being the mean fluorescence signal of the time points before ligand addition.

### Virus production

The biosensor AAV constructs were cloned in the Patriarchi laboratory and the opsin AAV construct was constructed by the VVF of the University of Zurich. All viral vectors were produced by the VVF. The viral titers of the viruses used in this study were AAVDJ.*hSynapsin1*.OxLight1 at 6.9 × 10^12^  GC ml^−1^; AAVDJ.*hSynapsin1*.OxLight-ctr at 1.1 × 10^13^ GC ml^−1^; AAV9.*hOX*.ChrimsonR.mCherry at 4.3 × 10^13^ GC ml^−1^; and AAV1.*hSynapsin1*.NES_jRCaMP1b at 6.9 × 10^12^ GC ml^−1^.

### Animals

Animal procedures were performed in accordance with the guidelines of the European Community Council Directive or the Animal Welfare Ordinance (TSchV 455.1) of the Swiss Federal Food Safety and Veterinary Office and were approved by the Zurich or Bern Cantonal Veterinary Office, the Government of Upper Bavaria or the National Council on Animal Care of the Italian Ministry of Health. Rat embryos (E17) obtained from timed-pregnant Wistar rats (Envigo) were used for preparing primary hippocampal neuronal cultures. Wild-type C57BL/6 mice (6–10 weeks old) of both sexes were used in this study. Mice were kept in cages with 1 to 5 individuals, at a temperature of approximately 22 °C and humidity level of about 40%, with ad libitum access to chow and water on either normal or reversed 12-h/12-h light/dark cycle for two-photon or photometry/optogenetic experiments, respectively. Optogenetic and behavior experiments were performed during the dark phase.

### Surgeries and viral injections

Surgeries were conducted under aseptic conditions on adult anesthetized mice (males and females, aged 6–10 weeks). Mice were anesthetized using isoflurane (initiation at 5% and maintenance at 0.8–2.5%).

For slice imaging experiments, AAVs encoding OxLight1 or OxLight-ctr (both 1:5) were unilaterally injected into the LH (1.5 mm anterior posterior (AP), 1.2 mm mediolateral (ML) and 5.15 mm dorsoventral (DV), volume 200 nl, rate 100 nl min^−1^). For combined photometry and optogenetic experiments as well as running behavior, AAVs encoding either OxLight1 or OxLight-ctr (both injected at a 1:5 dilution) were unilaterally injected into the NAcSh (1.5 mm AP, 1.05 mm ML and 4.83 mm DV, volume 300 nl, rate 100 nl min^−1^) and a commercially available 400-µm diameter optical fiber cannula (5 mm length, NA 0.57; Doric Lenses) was then implanted 0.1 mm above the injection site. The same mice also received bilateral injections of AAV9.*hOX*.ChrimsonR-mCherry into the LH at a volume of 250 nl for each site (injection rate, 100 nl min^−1^). A 200-µm diameter optical fiber cannula (NA 0.39; Thorlabs) was then placed 0.1 mm above the injection site during the same surgery. During surgery, a custom head plate was attached using dental cement (Herschel Dental). Animals received 5 mg kg^−1^ meloxicam injections for 3 d as postoperative pain medication. Experiments began 4 weeks after surgery.

For photometry experiments during tail-picking, mice were injected subcutaneously with an analgesic (metamizol at 200 mg kg^−1^ body weight) before surgery. Mice were injected unilaterally with 200 nl of either AAVDJ.*hSynapsin1*.OxLight1 or AAVDJ.*hSynapsin1*.OxLight-ctr into the posterior insular cortex (coordinates calculated from bregma were AP, −0.4 mm; ML, ±4.1 mm; and DV, −4.0 mm) at a rate of 75 μl min^−1^. Custom-made optic fibers (200-μm core diameter, 0.50 NA) glued to zirconia ferrules (2.5 mm) were implanted 0.15 mm above the viral injection site. Animals received subcutaneous carprofen (Rimadyl, Pfizer, 5 mg kg^−1^ body weight) for postoperative pain care for several days. Experiments started 4 weeks after surgery.

For sleep experiments, AAVs encoding OxLight1 or OxLight-ctr (both 1:1) mice were injected unilaterally in the LH (AP, −1.5 mm; ML, −0.9 mm; and DV, −5.35 mm) or BF (AP, +0.25 mm; ML, −1.3 mm; and DV, −5.4/−5.2 mm) using a microinfusion pump (Harvard Apparatus) and a 10-μl Hamilton syringe connected to a 29-gauge internal cannula. Viruses were infused with a flow rate of 0.1 μl min^−1^ and injection volume was 0.6 μl per injection site. One week following viral injections, a 400-µm optic fiber (NA 0.5; Thorlabs) housed in a stainless-steel ferrule (Thorlabs) was implanted using the same coordinates and fixed to the skull with Superbond C&B (Prestige Dental) and Paladur dental cement (Kulzer). A pair of stainless-steel screws (Paul Korth) were placed over the frontal and parietal cortices for EEG recordings. To monitor nuchal EMG activity two-wire electrodes (W3 Wire International) were inserted in the dorsal neck musculature. Electrodes were pre-soldered to an ultraminiature pin connector (Preci-Dip) and fixed to the skull with Superbond C&B (Prestige Dental) and Paladur dental cement (Kulzer).

For two-photon imaging experiments, AAVs encoding either OxLight1 or OxLight-ctr were injected in the somatosensory cortex. Analgesia was induced with a subcutaneous injection of dexamethasone (Dexadreson, 0.2 mg kg^−1^), topical anesthesia was delivered with an injection of 2% lidocaine under the scalp. A circular craniotomy (3 mm diameter) was centered at the stereotaxic coordinates corresponding to the right somatosensory cortex (−3.1 mm ML and −1.3 mm AP). A total volume of 300 nl of viral construct diluted in a 0.9% NaCl sterile solution (1:1) was slowly injected (70 nl min^−1^) into the cortical parenchyma through two injection sites. In the first site (−1.3 mm AP and −3.1 mm ML) we injected 50 nl virus 350, 300 and 250 µm below the pial surface. In the second site (approximately 700 µm posterior to the first one), we injected 50 nl 325, 275 and 225 µm below the surface. Injections were made using a pulled glass pipette (20–30 μm inner tip diameter) connected to a Hamilton syringe (VWR International) and an infusion pump. After injection, a custom chronic cranial window for two-photon imaging was placed above the craniotomy and secured with four drops of cyanoacrylate glue and dental cement (C&B Superbond; Sun Medical).

### Brain slice preparation, electrophysiology and imaging

For acute brain slice preparation, 4 weeks after viral injection 250-μm thick coronal brain slices were prepared after cervical dislocation and decapitation as previously described^35^. Patch clamp recordings and imaging were conducted in a slice chamber perfused with oxygenated aCSF containing, 126 mM NaCl, 3 mM KCl, 2 mM MgSO_4_, 2 mM CaCl_2_, 1.1 mM NaH_2_PO_4_, 26 mM NaHCO_3_, 0.1 mM pyruvic acid, 0.5 mM l-glutamine, 0.4 mM ascorbic acid and 25 mM d-glucose. To establish light responsiveness of ChrimsonR-expressing neurons, we recorded whole-cell currents during the application of light stimuli of different wavelengths. Briefly, 3–6 MOhm patch pipettes were filled with intracellular solution containing 130 mM potassium-gluconate, 5 mM NaCl, 2 mM MgSO_2_, 10 mM HEPES, 0.1 mM EGTA, 4 mM magnesium-ATP, 0.4 mM sodium-GTP, 2 mM pyruvic acid and ∼10 mM KOH. Whole-cell recordings were collected with HEKA EPC10 USB amplifiers and acquired with HEKA SmartLUX and Patchmaster v.2x90.5 software. Whole-cell membrane potential was recorded in a current clamp with zero holding current. Slices were subjected to illumination with either red laser light (20 Hz, 635 nm, 5 mW; Laserglow Technologies) or blue LED light (single-pulse, 465 nm, 1 mW) as used in photometry experiments (Doric Lenses). Light power output was measured with a power meter (Thorlab, PM100A). For establishing the ex vivo affinity of the sensor, indicated concentrations of OXA (Tocris) were prepared in aCSF and perfused on the slice chamber while the fluorescence of OxLight1 or OxLight-ctr was recorded using a Retiga ELECTRO camera (QImaging) using a 465 nm Sutter lambda 4DG as the light source, controlled by the HEKA Patchmaster. Images were acquired with HEKA SmartLUX and HEKA Patchmaster software. For establishing the ex vivo affinity of the sensor, indicated concentrations of OXA (Tocris) were prepared in aCSF at the indicated concentrations and perfused into the slice chamber for about 200 s. Data were analyzed and curves were fitted using Origin 2019b.

### In vivo photometry and optogenetics

Fluorescence from OxLight1 or OxLight-ctr were excited and detected at 100-μW LED light power at 465 nm wavelength with a Doric Fluorescence iFMC6_IE(400-410)_E1(460-490)_F1(500-540)_E2(555-570)_F2(580-680)_S filter cube and detectors. Emission signals of OxLight1 and OxLight-ctr were sampled at a frequency of 400 Hz with a photodetector fed into an I/O board (HEKA Patchmaster LIH 8+8). In optogenetic experiments, a red laser (635 nm; Laserglow Technologies) was connected bilaterally to LH optic fiber implants to yield ∼5 mW light power output at the fiber tip. Head-fixed mice were allowed to run freely on the wheel for 5 min, then a nozzle was fitted over their nose and mouth to induce anesthesia with 5% isoflurane for 1 min, then reduced to 2% isoflurane. In optogenetic experiments with variable frequencies, 5 min after anesthesia at 2% began, laser light was delivered at frequencies of either 1, 5, 10 or 20 Hz, with a pulse width of 5 ms and a train duration of 30 s. In optogenetic experiments with variable train duration, trains of light pulses were delivered on a different day at a frequency of 10 Hz for a duration of either 1, 5, 10, 15, 20 or 30 s and pulse width of 5 ms. In both experiment types, each train was repeated three times, delivered in a random order, with an inter-trial interval of 90 s. For analysis of optogenetic stimulation, custom-written code for MATLAB R2019b was used. Fluorescence traces were averaged in 100-ms bins and windows were made with a duration of 30 s before and 90 s after laser stimulus onset were selected. Traces were filtered using a Savitzky–Golay filter. Owing to changes in baseline values during anesthesia, traces were de-trended using a linear fit of the medians of the first and last 30 s of the 130-s sample window and then *z*-scored by each sample value having the baseline median subtracted from it and then being divided by the s.d. of the median baseline value. Baseline median and s.d. values were calculated using the 10-s window before stimulation. Peak stimulus values for each trial were determined by finding the maximum during the period that the laser was on. For rise times, windows from stimulus onset to 10 s were selected and smoothed with a Savitzky–Golay filter with a length of 4 s. Smoothed traces were then fitted with a one-phase exponential association model using custom-written MATLAB code. For decay times, windows from stimulus offset to the minimum value detected after stimulus offset were selected, smoothed and fitted as above. Fits that were too poor were excluded, resulting in five traces being removed in total.

### Spontaneous running experiments

Mice were head-fixed and allowed to run on a treadmill consisting of a disk attached to a rotary encoder that output a state change signal every 1 degree of rotation. Mice were placed at a distance of about 11 cm from the center of the wheel, so that the distance walked with each state change was around 200 mm. Photometry was recorded as described above. Running and fluorescence traces were binned into 100-ms bins. Running bout onsets were detected as at least 65 state changes (13 cm) made within 5 s following a quiescent period of 1 s when one or no state changes had occurred. Values were calculated as the maximum running speed or maximum fluorescence (*z*-scored as above, but not de-trended) taken from a period 2.5–5 s after spontaneous running bout onset and pooled values were expressed as mean ± s.e.m.

### Tail-picking experiments

Mice were handled by the experimenter for 5 d for around 5 min and habituated to being tethered to the optic patch cords for 3 d before behavioral procedures. All behavioral experiments were performed during the dark phase of the light cycle. Mice were placed inside a custom cylindrical chamber (30-cm diameter) and were free to explore it for 4 min. At the end of the experiment, mice were picked up by the base of their tail by the experimenter and were held 30 cm above the ground for approximately 10–15 s. Photometry recordings were synchronized with ANYmaze software v.6.05 (64-bit, Stoelting) using transistor-to-transistor logic pulses. Videos of the behavioral experiments were manually scored by the experimenter. Photometry data were analyzed using custom-written Python code. Mean event-related Δ*F* / *F*_0_ changes in OxLight1 or OxLight-ctr signals were normalized over the s.d. calculated 5 s before the start of each event. Values for the pre-event (5 s before tail lifting) and event (10 s of tail lifting) were computed and compared using a two-sided paired Student’s *t*-test.

### Polysomnographic recordings

One week following implantation surgeries, mice were connected to flexible cables and patch cords for an additional 7 d of habituation to the recording conditions. During the experimental sessions, fluorescence signals were paired with polysomnographic recordings and recorded for 2 h. Fluorescence excitation was obtained with 470 nm LEDs (Thorlabs). Signals were normalized to provide ∆*F*/*F*_0_ values using custom-written MATLAB codes. EEG and EMG signals were amplified (A-M systems) and digitized at 512 Hz using custom-written scripts in Labview 2019. Polysomnographic recordings were visually scored by 1-s epochs using custom-made MATLAB code. Vigilance states were classified as wake, NREM sleep and REM sleep based on analyses of EEG and EMG recordings. Wake was characterized by a low amplitude, mixed-frequency EEG signal in association with a relatively elevated and variable EMG muscle tone and activity. NREM sleep was defined by an EEG showing synchronous high-amplitude slow-wave activity in the δ frequency range (0.5–4 Hz) with a low and stable muscle tone. REM sleep was characterized by ϑ oscillations (6–9 Hz) and a neck muscle atonia. Suvorexant tablets (Belsomra, 10 mg, Merck) was reduced into a fine powder using a pharmaceutical grade crusher. The powder was weighed and mixed in NaCl (0.9%) to a volume of 10 μl g^−1^ body weight to reach the desired dose (50 mg kg^−1^), which was then administered by oral gavage (per os) to mice. Suvorexant administration was followed by fiber photometry and sleep recordings for 3–4 h. For photometry during anesthesia, imaging and sleep recordings were performed continuously before, during and after both isoflurane and medetomidine administration. Tethered animals were put in a movable custom-designed anesthesia box and were anesthetized with isoflurane (2% in 1 l m^−1^ O_2_) for 10 min. After this time, they were moved back to their home cage. Medetomidine (Dorbene, 0.5 mg kg^−1^) mixed in NaCl (0.9%) to a volume of 3 μl g^−1^ body weight was administered by intraperitoneal injection to mice. After 30 min, anesthesia was reversed by subcutaneous injection of atipamezole (Alzane, 2.5 mg kg^−1^) mixed in 0.5 ml NaCl (0.9%) to a volume of 9 μl g^−1^ body weight. This led to a clear reversal of anesthesia within a few minutes.

### In vivo two-photon imaging

Two-photon imaging was performed using a Chameleon Discovery pulsed laser (80 MHz pulse frequency, Coherent Inc.) tuned at 920 nm using Prairie View 5.4 software. Excitation power was controlled via a Pockel cell (Conoptics) and was 65–98 mW under the microscope objective. An Ultima II scanhead (Bruker Corporation) was equipped with 3-mm raster scanning galvanometers (6215H, Cambridge Technology). A ×16/0.80W objective (Nikon) was used and emitted photons went through a 525/50 filter before reaching a GaAsP photomultiplier (Hamamatsu Photonics). Dwell time was between 3.6 and 4 μs, photomultiplier voltage was 790 V, zoom factor was always 1, acquisition frame rate ranged between 4.9 and 7.5 Hz, depending on the size of the ROI selected for imaging. Pixel size was always 1.27 μm. Mice were handled for 5 to 7 d before the first day of imaging to familiarize them with the experimenter and with head fixation. Mice were habituated to voluntarily enter and rest in a plastic tube, where they subsequently rested and were head-fixed during imaging. Anesthesia was induced through a nose mask using 3% isoflurane in 1 l min^−1^ O_2_ for 1 min. The anesthetized state was maintained (1–1.3% isoflurane in 1 l min^−1^ O_2_) for 13 to 17 min. Once imaging acquisition started, the anesthetic regime was maintained for 1 min more, after which the isoflurane setup was switched off and the mouse was allowed to wake up. Imaging continued for another 5 to 7 min after isoflurane delivery was turned off. OxLight1 activity was imaged during wakefulness in two mice. Awake recordings started 13 min after isoflurane delivery was stopped and lasted 11 min.

### Data analysis for two-photon recordings

Imaging videos were imported into the Suite2p (v.0.10.2) two-photon software through its Python 3.9.2 graphical user interface and mechanical drift correction was automatically performed^52^. The resulting tiff images were imported into MATLAB for further analysis on the basis of custom scripts. To compare orexin activity in OxLight1 and OxLight-ctr mice during anesthesia, we identified an ‘anesthetized period’, corresponding to the frames acquired during the first minute of imaging (when isoflurane was delivered). For the analysis in Fig. 6c–e and Extended Data Fig. 10c,f, each imaging frame was treated as one unique ROI, containing all the pixels in the image. Signal at each time frame was calculated as the average fluorescence across all pixels inside this ROI (*F*(*t*)). When comparing orexin activity between the first and last minute of imaging (Fig. 6e), the baseline fluorescence (*F*_0_) was calculated as the median of the fluorescence values distribution across all frames acquired during the anesthetized period. The fluorescence time series for each FOV was then corrected for the baseline using the formula (*F*(*t*) – *F*_0_) / *F*_0_ (referred to as Δ*F* / *F*_0_).

To compare orexin activity in OxLight1 and OxLight-ctr mice during emergence from anesthesia, we limited our analysis to a period between the third minute of imaging (1 min after switching isoflurane off) and the sixth minute of imaging. During this period, we identified the 1-min-long time window carrying the highest fluorescence variance across frames. We repeated this process for each individual FOV. To identify this time window, we calculated the s.d. of the distribution of pixel values for each frame of a moving window extending for *n* frames, with *n* being the number of frames acquired in 1 min of imaging for each given FOV. We then computed the average s.d. across the *n* frames of each moving window and selected the window with the highest average s.d. as the one representing the 1 min of highest activity (referred to as ‘most-active minute’). All the following analysis was run on this time window. To identify ROIs in each field of view, we first binarized the FOV during this 1-min-long window. Briefly, we assigned a value of 1 or 0 to every pixel (i;j) in every frame (*t*) acquired during the last minute of imaging, according to the following:

$$b(i;j;t) = \left\{ {\begin{array}{*{20}{l}} {1,} \hfill & {f(i;j;t) > {\mathrm{median}}(f(i;j;{\Delta}t)) + {\mathrm{s.d.}}(f(i;j;{\Delta}t))} \hfill \\ {0,} \hfill & {\mathrm{otherwise}} \hfill \end{array}} \right.$$

with *b* being the binarized activity, *f* the fluorescence value of every pixel at every frame (*t*) and Δ*t* encompassing the 1 min of imaging with highest variance. Then, we applied a two-dimensional Gaussian filter to each frame of the FOV using the MATLAB function imfilter (s.d. of the filter was 2 pixels). This operation resulted in a smoothened version of the binarized frames, with each pixel having a value between 0 and 1. Finally, we deconvolved each frame using the MATLAB function deconvlucy. The size of the two-dimensional Gaussian filter was 50 µm × 50 µm (39 × 39 pixels) and the s.d. was 20 pixels. To identify ROIs in each FOV we proceeded as follows. We obtained an average projection image for each deconvolved FOV. The pixel values obtained through the deconvolution process are comparable across FOVs. Therefore, we computed the distribution of pixel values across all 16 projected and deconvolved FOVs (8 OxLight1 and 8 OxLight-ctr). We then identified, for each FOV, ten circular ROIs (diameter 12 pixels, 20.4 µm) containing pixels with values between the 70th and the 100th percentile of the aforementioned distribution. These ROIs are referred to as ‘active ROIs’. Moreover, we identified ten further ROIs of the same size and shape, including pixels with values between the 5th and the 69th percentile of the distribution (inactive ROIs). Pixel values below the fifth percentile of the distribution were always excluded from the ROIs, as these corresponded to blood vessels running perpendicular to the brain surface.

Throughout the analysis, we tested the hypothesis that data came from a normally distributed population using the lillietest function in MATLAB.

### Immunohistochemistry

Mice were killed with an intraperitoneal injection of pentobarbital (>150 mg kg^−1^) and transcardially perfused with a PBS solution containing 4% PFA. Brains were post-fixed for 18 h in the same solution and then transferred to a solution of 30% sucrose in PBS overnight. Coronal brain slices with 40–60 μm thickness were then prepared using a cryostat and 40-µm thick coronal sections were obtained using a freezing stage microtome (microm HM450; Thermo Scientific). For immunostaining, slices were first rinsed three times with washing solution (0.3% Triton X-100 in PBS), then incubated with blocking solution (1% BSA in PBS) for 1 h. Chicken anti-GFP (1:500 dilution; Abcam, cat. no. ab13970), rabbit anti-mCherry (1:500 dilution; Abcam, cat. no. ab183628) or mouse anti-OXA (1:500 dilution; Santa Cruz Biotechnology, cat. no. sc80263) primary antibodies were then added to the blocking solution for 24 h at 4 °C, followed by three washes with washing solution and incubation with either Alexa-488-labeled goat anti-chicken (1:1,000 dilution, Abcam, cat. no. ab150169), Alexa-488-labeled goat anti-mouse (1:1,000 dilution, Thermo Fisher, cat. no. A32723), Alexa-568-labeled goat anti-mouse (1:500 dilution, Thermo Fisher, cat. no. A-11004) or Alexa-546-labeled goat anti-rabbit (1:1,000 dilution, Thermo Fisher, cat. no. A11035) in PBS for 2 h at room temperature. Some sections were finally stained for 20 min with Hoechst 33342 (Molecular Probes; 1:300 dilution in PBS) and mounted or mounted with Fluoromount-G mounting medium containing DAPI (Invitrogen). Images were acquired on a Fluoview 3000 confocal microscope (Olympus).

### Statistical analysis

For pairwise analysis of sensor variants, the statistical significance of their responses was determined on a case-by-case basis using a two-tailed unpaired Student’s *t*-test with Welch’s correction. A Brown–Forsythe ANOVA test followed by Dunnett’s T3 multiple comparison was used for comparison of more than two datasets. For in vivo imaging experiments, we used paired and unpaired Student’s *t*-tests for statistical analysis of parametric data and Wilcoxon rank-sum or signed-rank tests for analysis of nonparametric data. Immunohistochemistry (IHC) experiments were repeated at least twice with similar results. All *P* values are indicated in figure legends. Data are displayed as mean with s.e.m. No statistical methods were used to predetermine sample size.

### Reporting Summary

Further information on research design is available in the Nature Research Reporting Summary linked to this article.

## Online content

Any methods, additional references, Nature Research reporting summaries, source data, extended data, supplementary information, acknowledgements, peer review information; details of author contributions and competing interests; and statements of data and code availability are available at 10.1038/s41592-021-01390-2.

## Supplementary information

Supplementary Information Supplementary Note and Supplementary Figs. 1–3.

Reporting Summary

Supplementary Video 1 Two-photon movie of an FOV acquired in the somatosensory cortex of a mouse expressing OxLight1 in layer 2/3. Isoflurane anesthesia was on for the first minute of imaging and was then turned off while the mouse was allowed to recover. Scale bar, 50 μm. Images were acquired at 5 Hz.

Supplementary Video 2 Two-photon movie of an FOV acquired in the somatosensory cortex of a mouse expressing OxLight-ctr in layer 2/3. Isoflurane anesthesia was on for the first minute of imaging and was then turned off while the mouse was allowed to recover. Scale bar, 50 μm. Images were acquired at 5 Hz.

## Data Availability

DNA and protein sequences of the sensors developed in this study have been deposited on the National Center for Biotechnology Information database (accession nos. MW845970 and MW845971) and are available in the Supplementary Note. The corresponding DNA plasmids for viral production have been deposited both at the VVF (https://vvf.ethz.ch/) and at Addgene. Viral vectors for sensor expression can be obtained either from the Patriarchi laboratory, the VVF or Addgene. All source data are provided with the manuscript. All other raw data can be made available upon reasonable request. Accession codes (Protein Data Bank) are 5WQC, 3SG7, 1WSO and 1CQ0. Source data are provided with this paper.

## Source data

Source Data Fig. 1 Source data from the development of the orexin sensor.
Source Data Fig. 2 Source data from the sensor characterization in vitro.
Source Data Fig. 3 Source data from the ex vivo and in vivo validation of the sensor.
Source Data Fig. 4 Source data from the monitoring orexin dynamics associated with natural behaviors.
Source Data Fig. 5 Source data from the tracking of orexin dynamics across sleep–wake cycles.
Source Data Fig. 6 Source data from the two-photon imaging of cortical orexin dynamics during emergence from anesthesia.
Source Data Extended Data Fig. 1 Source data from the optimization of cpGFP insertion site into OX2R.
Source Data Extended Data Fig. 2 Source data from the optimization of OxLight0.1 dynamic range.
Source Data Extended Data Fig. 3 Source data from the development and characterization of a control sensor.
Source Data Extended Data Fig. 4 Source data from the spectral properties of OxLight1.
Source Data Extended Data Fig. 5 Source data from the brightness and pH sensitivity of OxLight1.
Source Data Extended Data Fig. 6 Source data from the additional characterization of OxLight1.
Source Data Extended Data Fig. 7 Source data from the characterization of sensor coupling to intracellular signaling partners.
Source Data Extended Data Fig. 8 Source data from the dynamic orexin fluctuations during brain states.
Source Data Extended Data Fig. 10 Source data from the characterization of OxLight1 and OxLight-ctr expression in somatosensory cortex.

## Code availability

Custom MATLAB and Python code used for data processing and analysis is available at https://github.com/patriarchilab/OxLight1. All other custom code can be made available upon reasonable request.
