# Supplementary material for: A genetically encoded sensor for in vivo imaging of orexin neuropeptides
Source: Nat Methods. 2022 Feb 10;19(2):231–41. doi: 10.1038/s41592-021-01390-2 (PMC8831244; doi:10.1038/s41592-021-01390-2)
Supplement: Supplementary file 1 — Supplementary Note and Supplementary Figs. 1–3. [file 41592_2021_1390_MOESM1_ESM.pdf]

nature portfolio

https://doi.org/10.1038/s41592-021-01390-2

Supplementary information

A genetically encoded sensor for in vivo imaging of orexin neuropeptides

In the format provided by the authors and unedited

Supplementary Note | Sequences of sensor constructs generated in this study.

OxLight1 DNA sequence

atgaagacgatcatcgccctgagctacatcttctgcctggtgttcgccgactacaaggacgatgatgacgccatgtcaggcacaa
aactggaagactcccctccttgtcggaattggtcctccgcgtccgagcttaacgaaacgcaagaaccttttcttaatccgacagact
acgatgatgaagagttccttaggtatctctggagggagtatctgcatcccaaagaatatgaatgggtactgatcgcgggatatatc
attgtctttgtggtggccctgattgggaatgtactcgtatgtgtagcagtttggaaaaaccatcatatgagaactgttaccaactattta
ttgtaaacctgagccttgcagacgtgttggtgacaatcacgtgcctcccggctaccctcgttgttgacataaccgagacatggttctt
cggtcaaagtctgtgtaaggtgataccatatctccagaccgtgagcgtttctgtgagcgtgcttactcttagctgtatagctctcgatc
gctggtacgcgatatgtcaccctcacaagttcaagagtacggctaagagggctaggaattctatagtcattatttggatcgtgtcat
gcataataatgataccgcaggcgattgtcatggaatgctctacggtatttccgggtttggcaaataaaacgacgttgtttacagtttg
cgacgagagatggggtggggagatttatccgaagatgtatcacatatgtttcttcctcgtcacttatatggctccgctctgcttgatggt
cctggcgtaccttcaaatctttcggaaactgtggtgccgagagttgtccagtctcatcaatgtctacataaaggcggataaacaaa
aaaacggaattaaagcaaatttcaagataagacacaacattgaagacggtggcgttcagcttgcatatcattaccaacagaata
cccccatcggcgacggtccggtgcttctgccagacaaccattacctgtccgtacaatcaaagttgtccaaagaccctaatgagaa
acgcgaccatatggtactgcttgaattcgtgaccgcagcaggaataactctgggcatggatgaactctataagggaggaacag
gtggatcaatggtatcaaagggcgaggaacttttcacaggagtcgtcccaattcttgttgagttggacggcgacgttaatggacac
aagttttccgtcagtggcgagggagagggcgacgctacatatgggaagttgacgctcaaattcatatgtactactggtaagctgc
ctgtaccttggccgactttggtcactaccttgacctatggtgttcagtgtttcagcagatacccggaccatatgaagcagcacgattttt
ttaaaagtgccatgcccgaaggttatattcaagagcgcactatcttcttcaaggacgacgggaactataagacgcgggctgaggt
aaagttcgagggagatacattggtcaatcgaattgagcttaaagggatcgacttcaaagaagacggaaacatcttgggacaca
agctggagtataataatcatgaccagttgagacagattcgcgcgaggcggaaaacggcacggatgcttatgatcgttcttcttgta
ttcgctatatgctatctccctatctccattcttaacgttttgaaaagagtgttcggtatgttcgcacacacggaagatagagaaactgtg
tacgcgtggttcactttcagccattggctggtctacgccaacagcgcagcgaatcctataatctacaatttcctttcaggcaagtttcg
agaggaatttaaggctgcgttttcctgttgctgcctgggtgttcaccatcggcaagaagatcgactcacacgaggtcgaacgtcaa
ctgaaagcagaaagtctcttacgacacaaatcagcaattttgataacattagcaaactgtccgaacaggttgtcttgaccagcatc
agtaccttgcccgccgcaaacggcgccggccctcttcagaattggtga

OxLight1 protein sequence

MSGTKLEDSPPCRNWSSASELNETQEPFLNPTDYDDEEFLRYLWREYLHPKEYEWVLIAG
YIIVFVVALIGNVLVCVAVWKNHHMRTVTNYFIVNLSLADVLVTITCLPATLVVDITETWFFGQ
SLCKVIPYLQTVSVSVSVLTLSCIALDRWYAICHPHKFKSTAKRARNSIVIIWIVSCIIMIPQAIV
MECSTVFPGLANKTTLFTVCDERWGGEIYPKMYHICFFLVTYMAPLCLMVLAYLQIFRKLWC
RELSSLINVYIKADKQKNGIKANFKIRHNIEDGGVQLAYHYQQNTPIGDGPVLLPDNHYLSVQ
SKLSKDPNEKRDHMVLLEFVTAAGITLGMDELYKGGTGGSMVSKGEELFTGVVPILVELDG
DVNGHKFSVSGEGEGDATYGKLTLKFICTTGKLPVPWPTLVTTLTYGVQCFSRYPDHMKQ
HDFFKSAMPEGYIQERTIFFKDDGNYKTRAEVKFEGDTLVNRIELKGIDFKEDGNILGHKLEY
NNHDQLRQIRARRKTARMLMIVLLVFAICYLPISILNVLKRVFGMFAHTEDRETVYAWFTFSH
WLVYANSAANPIIYNFLSGKFREEFKAAFSCCCLGVHHRQEDRLTRGRTSTESRKSLTTQIS
NFDNISKLSEQVVLTSISTLPAANGAGPLQNW*

OxLight-ctr DNA sequence

atgaagacgatcatcgccctgagctacatcttctgcctggtgttcgccgactacaaggacgatgatgacgccatgtcaggcacaa
aactggaagactcccctccttgtcggaattggtcctccgcgtccgagcttaacgaaacgcaagaaccttttcttaatccgacagact
acgatgatgaagagttccttaggtatctctggagggagtatctgcatcccaaagaatataaatgggtactgatcgcgggatatatc
attgtctttgtggtggccctgattgggaatgtactcgtatgtgtagcagtttggaaaaaccatcatatgagaactgttaccaactatttta
ttgtaaacctgagccttgcagacgtgttggtgacaatcacgtgcctcccggctgccctcgttgttgacataaccgagacatggttctt
cggtcaaagtctgtgtaaggtgataccatatctccagaccgtgagcgtttctgtgagcgtgcttactcttagctgtatagctctcgatc
gctggtacgcgatatgtcaccctcacaagttcaagagtacggctaagagggctaggaattctatagtcattatttggatcgtgtcat
gcataataatgataccgcaggcgattgtcatggaatgctctacggtatttccgggtttggcaaataaaacgacgttgtttacagtttg
cgacgagagatggggtggggagatttatccgaagatgtatcacatatgtttcttcctcgtcacttatatggctccgctctgcttgatggt
cctggcgtaccttcaaatctttcggaaactgtggtgccgagagttgtccagtctcatcaatgtctacataaaggcggataaacaaa
aaaacggaattaaagcaaatttcaagataagacacaacattgaagacggtggcgttcagcttgcatatcattaccaacagaata
cccccatcggcgacggtccggtgcttctgccagacaaccattacctgtccgtacaatcaaagttgtccaaagaccctaatgagaa
acgcgaccatatggtactgcttgaattcgtgaccgcagcaggaataactctgggcatggatgaactctataagggaggaacag
gtggatcaatggtatcaaagggcgaggaacttttcacaggagtcgtcccaattcttgttgagttggacggcgacgttaatggacac
aagttttccgtcagtggcgagggagagggcgacgctacatatgggaagttgacgctcaaattcatatgtactactggtaagctgc
ctgtaccttggccgactttggtcactaccttgacctatggtgttcagtgtttcagcagatacccggaccatatgaagcagcacgattttt
ttaaaagtgccatgcccgaaggttatattcaagagcgcactatcttcttcaaggacgacgggaactataagacgcgggctgaggt
aaagttcgagggagatacattggtcaatcgaattgagcttaaagggatcgacttcaaagaagacggaaacatcttgggacaca
agctggagtataataatcatgaccagttgagacagattcgcgcgaggcggaaaacggcacggatgcttatgatcgttcttcttgta
ttcgctatatgctatctccctatctccattcttaacgttttgaaaagagtgttcggtatgttcgcacacacggaagatagagaaactgtg
tacgcgtggttcactttcagccattggctggtctacgccaacagcgcagcgaatcctataatctacaatttcctttcaggcaagtttcg
agaggaatttaaggctgcgttttcctgttgctgcctgggtgttcaccatcggcaagaagatcgactcacacgaggtcgaacgtcaa
ctgaaagcagaaagtctcttacgacacaaatcagcaattttgataacattagcaaactgtccgaacaggttgtcttgaccagcatc
agtaccttgcccgccgcaaacggcgccggccctcttcagaattggtga

OxLight-ctr protein sequence

MSGTKLEDSPPCRNWSSASELNETQEPFLNPTDYDDEEFLRYLWREYLHPKEYKWVLIAG
YIIVFVVALIGNVLVCVAVWKNHHMRTVTNYFIVNLSLADVLVTITCLPAALVVDITETWFFGQ
SLCKVIPYLQTVSVSVSVLTLSCIALDRWYAICHPHKFKSTAKRARNSIVIIWIVSCIIMIPQAIV
MECSTVFPGLANKTTLFTVCDERWGGEIYPKMYHICFFLVTYMAPLCLMVLAYLQIFRKLWC
RELSSLINVYIKADKQKNGIKANFKIRHNIEDGGVQLAYHYQQNTPIGDGPVLLPDNHYLSVQ
SKLSKDPNEKRDHMVLLEFVTAAGITLGMDELYKGGTGGSMVSKGEELFTGVVPILVELDG
DVNGHKFSVSGEGEGDATYGKLTLKFICTTGKLPVPWPTLVTTLTYGVQCFSRYPDHMKQ
HDFFKSAMPEGYIQERTIFFKDDGNYKTRAEVKFEGDTLVNRIELKGIDFKEDGNILGHKLEY
NNHDQLRQIRARRKTARMLMIVLLVFAICYLPISILNVLKRVFGMFAHTEDRETVYAWFTFSH
WLVYANSAANPIIYNFLSGKFREEFKAAFSCCCLGVHHRQEDRLTRGRTSTESRKSLTTQIS
NFDNISKLSEQVVLTSISTLPAANGAGPLQNW*

Labeled in green is the cpGFP module.

Highlighted in magenta are the E54$^{1.32}$K and T111$^{2.61}$A mutations used in the control sensor.

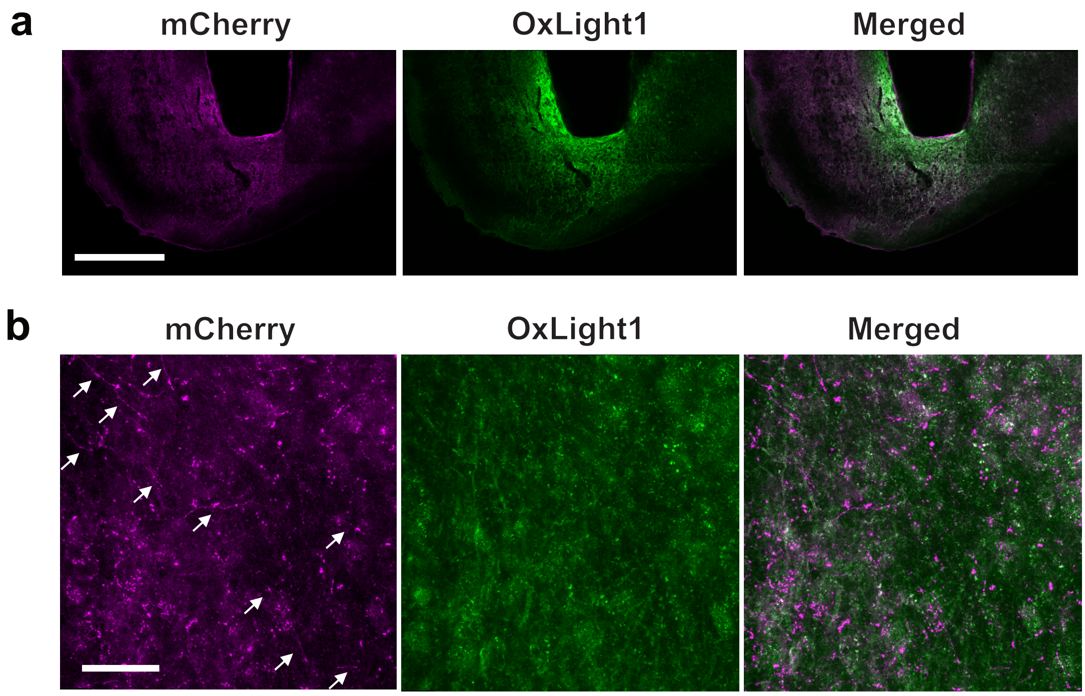

Supplementary Figure 1 | Immunohistochemical verification of virus expression in NAc
a, Low magnification (20X) immunohistochemical visualization of ChrimsonR-mCherry (anti-mCherry labeling, magenta) and OxLight1 (anti-GFP labeling, green) expression in the NAcSh below the location of optic fiber tip. Scale bar, 500 µm. **b**, Higher magnification images from the same sample in **a** (60X). Arrows indicate orexinergic fibers. Scale bar, 50 µm.

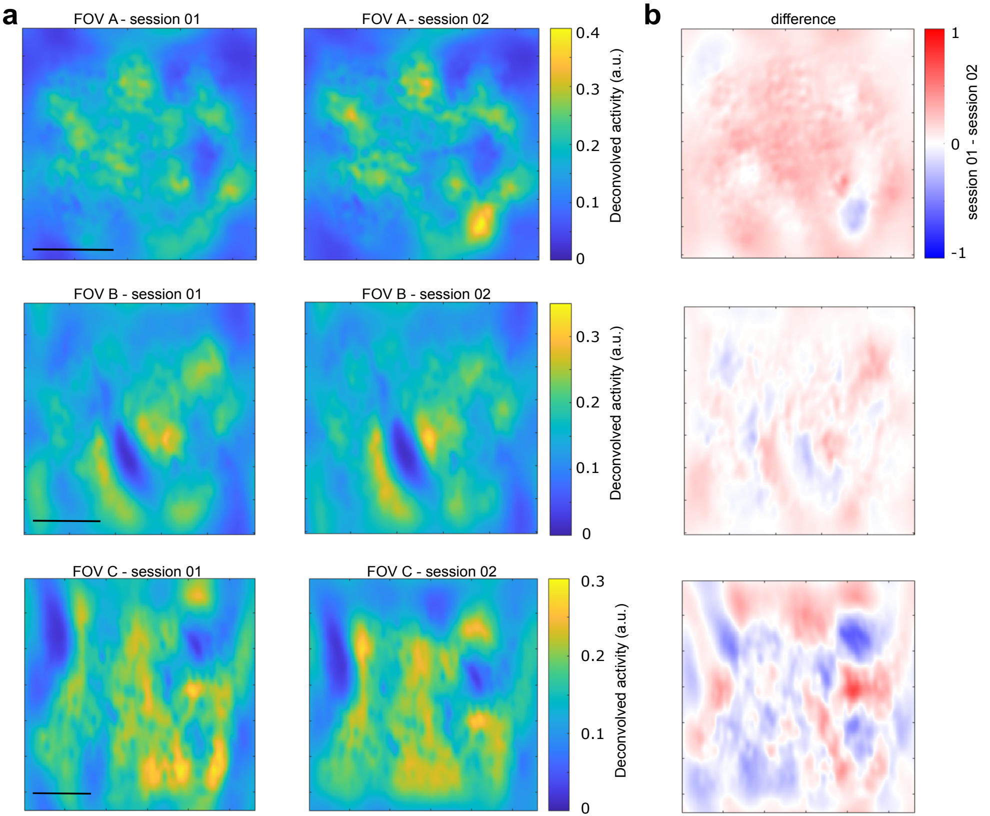

Supplementary Figure 2 | Localized OxLight1 activity across multiple imaging sessions of the same FOV
a, left: Average projection of deconvolved OxLight1 activity in one FOV (FOV A - session 01) imaged during awakening from 15 minutes of isoflurane anesthesia. Middle: same as in left, for the same FOV imaged during awakening from a second 15-minutes cycle of isoflurane delivery (FOV A - session 02). Right: pixel-by-pixel difference between FOV A - session 01 and FOV A - session 02. **b**, **c**, same as in **a** for two further FOVs (FOV B and FOV C, respectively). Scale bar is always 100 um. The average correlation coefficient between the two projections was 0.769+/-0.224 (average across the 3 FOVs, +/- s.d.).

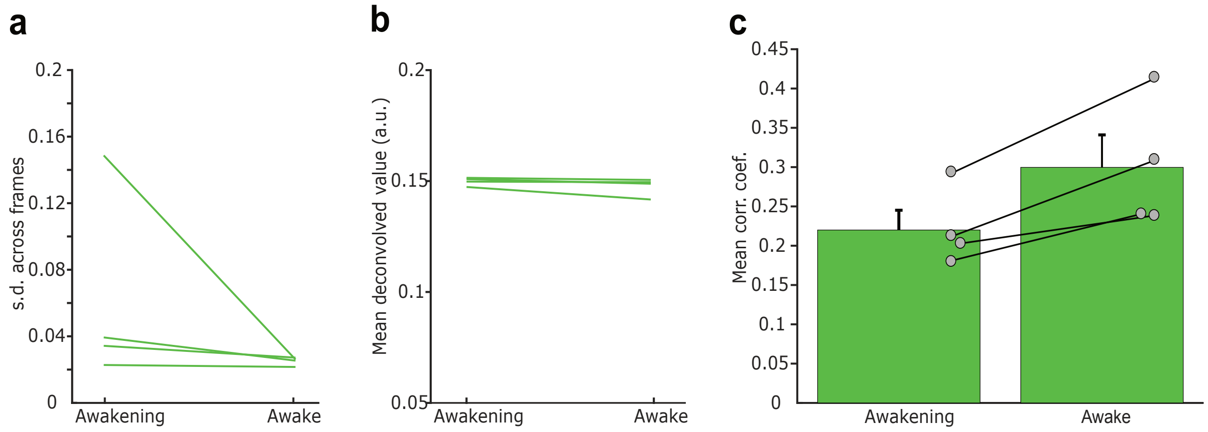

Supplementary Figure 3 | Localized OxLight1 activity during awake recordings, related to Extended Data Figure 10 of the article.
a, Mean standard deviation of the average fluorescence value calculated across frames during a 1-minute long time window identified as the “most active minute”. Left data points show values for FOVs recorded while OxLight1 expressing mice were emerging from anesthesia. Data on the right report values from the same FOVs, but recorded during awake periods (one-sided paired t-test, p = 0.299; Wilcoxon rank-sum, p = 0.2). **b**, Mean deconvolution value obtained for 4 FOVs recorded during emergence from anesthesia (left) and the same FOVs recorded while mice were fully awake (right). (one-sided paired t-test, p = 0.158; Wilcoxon rank-sum, p = 0.342). **c**, Pearson's correlation coefficients between all active ROI pairs in 4 example OxLight1 FOVs, computed while mice were emerging from anesthesia (left) and when mice were fully awake (right) (mean +/- SEM). Correlation coefficients were always computed during the most active minute of imaging. Individual data points are shown as grey dots. (paired one-sided t-test, p = 0.021; Wilcoxon rank-sum, p = 0.114).
