# Supplementary material for: A genetically encoded sensor for in vivo imaging of orexin neuropeptides
Source: Nat Methods. 2022 Feb 10;19(2):231–41. doi: 10.1038/s41592-021-01390-2 (PMC8831244; doi:10.1038/s41592-021-01390-2)
Supplement: Supplementary file 2 — Reporting Summary [file 41592_2021_1390_MOESM2_ESM.pdf]

nature research

Corresponding author(s): Tommaso Patriarchi

Last updated by author(s): October 27, 2021

Reporting Summary

Nature Research wishes to improve the reproducibility of the work that we publish. This form provides structure for consistency and transparency in reporting. For further information on Nature Research policies, see our Editorial Policies and the Editorial Policy Checklist.

Statistics

For all statistical analyses, confirm that the following items are present in the figure legend, table legend, main text, or Methods section.

n/a	Confirmed	
☐	☒	The exact sample size (*n*) for each experimental group/condition, given as a discrete number and unit of measurement
☐	☒	A statement on whether measurements were taken from distinct samples or whether the same sample was measured repeatedly
☐	☒	The statistical test(s) used AND whether they are one- or two-sided<br>*Only common tests should be described solely by name; describe more complex techniques in the Methods section.*
☒	☐	A description of all covariates tested
☐	☒	A description of any assumptions or corrections, such as tests of normality and adjustment for multiple comparisons
☐	☒	A full description of the statistical parameters including central tendency (e.g. means) or other basic estimates (e.g. regression coefficient) AND variation (e.g. standard deviation) or associated estimates of uncertainty (e.g. confidence intervals)
☐	☒	For null hypothesis testing, the test statistic (e.g. *F*, *t*, *r*) with confidence intervals, effect sizes, degrees of freedom and *P* value noted<br>*Give P values as exact values whenever suitable.*
☒	☐	For Bayesian analysis, information on the choice of priors and Markov chain Monte Carlo settings
☒	☐	For hierarchical and complex designs, identification of the appropriate level for tests and full reporting of outcomes
☒	☐	Estimates of effect sizes (e.g. Cohen's *d*, Pearson's *r*), indicating how they were calculated

Our web collection on statistics for biologists contains articles on many of the points above.

Software and code

Policy information about availability of computer code

Data collection	Below is the software used for data collection in this manuscript:<br>• Zeiss Zen Blue 2018 (v2.6) (https:/ /www.zeiss.com/microscopy/int/products/microscope-software/zen-lite.html)<br>• Scanimage 5 (http://scanimage.vidriotechnologies.com/display/51 H/Scan Image+Home)<br>• HEKA SmartLUX and Patchmaster v2x90.5 (https://www.heka.com/)<br>• Labview 2019 (https://www.ni.com/en-us/support/downloads/software-products/download.labview.html)<br>• Prairie View 5.4 (https://www.bruker.com/en/products-and-solutions/fluorescence-microscopy/multiphoton-microscopes.html)<br>• Suite2p v0.10.2 (https://github.com/MouseLand/suite2p)<br>• ANY-maze Video Tracking System version 6.05 (64-bit) (https://www.stoeltingco.com/anymaze.html)
Data analysis	Below is the software used for data analysis in this manuscript:<br>• Molecular Operating Environment 2020 (https://www.chemcomp.com/Products.htm)<br>• ImageJ version 1.52 (http://imagej.nih.gov/ij/download.html)<br>• GraphPad Prism 9.0.0 (https://www.graphpad.com/scientific-software/prism/)<br>• MATLAB version R2019a (https://www.mathworks.com/products/matlab.html)<br>• MATLAB version R2019b (https://www.mathworks.com/products/matlab.html)<br>• Origin 2019b (https://www.originlab.com/origin)<br>• Python 3.9.2 (https://www.python.org/downloads/)<br>Custom code is available on https://github.com/patriarchilab/OxLight1.

For manuscripts utilizing custom algorithms or software that are central to the research but not yet described in published literature, software must be made available to editors and reviewers. We strongly encourage code deposition in a community repository (e.g. GitHub). See the Nature Research guidelines for submitting code & software for further information.

Data

Policy information about availability of data

All manuscripts must include a data availability statement. This statement should provide the following information, where applicable:

- Accession codes, unique identifiers, or web links for publicly available datasets
- A list of figures that have associated raw data
- A description of any restrictions on data availability

DNA and protein sequences for the sensors developed in this study were deposited on NCBI (accession numbers MW845970 - MW845971) and are available in Supplementary Data S1. The corresponding DNA plasmids for viral production have been deposited both on the UZH Viral Vector Facility (https://vvf.ethz.ch/) and on Addgene. Viral vectors for sensor expression can be obtained either from the Patriarchi laboratory, the UZH Viral Vector Facility, or Addgene. All source data are provided with the manuscript. Raw data can be made available upon request. Accession codes (Protein Data Bank) PDBIDs: 5WQC, 3SG7, 1WSO, 1CQ0.

Field-specific reporting

Please select the one below that is the best fit for your research. If you are not sure, read the appropriate sections before making your selection.

☒ Life sciences ☐ Behavioural & social sciences ☐ Ecological, evolutionary & environmental sciences

For a reference copy of the document with all sections, see nature.com/documents/nr-reporting-summary-flat.pdf

Life sciences study design

All studies must disclose on these points even when the disclosure is negative.

Sample size	No sample size calculation was performed. Sample sizes were based on the previous scientific literature in the field [Patriarchi T. et al., Science 360, 6396; Patriarchi T. et al, Nat Methods 17, 1147-1155; Sun F. et al, Cell 174, 481-496; Sun F. et al, Nat Methods 17, 1156-1166].
Data exclusions	During in vitro experiments for determining sensor kinetics, line-scans with latencies of co-applied red dye fluorescence signal onset above 50 ms were excluded from analysis. For rise or decay time calculation from optogenetic experiments, fits that were too poor were excluded, resulting in 5 traces being removed in total.
Replication	For cell culture work, all experiments where statistical analysis was performed were repeated at least three times. All experiments involving animals were repeated at least twice. The exact number of animals used per in vivo experiment is reported in the respective figure legend. All replication attempts were successful.
Randomization	Group allocations used in this study were randomly assigned to animals and/or cultured cells.
Blinding	Blinding was not necessary and therefore was not adopted in this study, due to the fact that we only tested one sensor (OxLight1).

Reporting for specific materials, systems and methods

We require information from authors about some types of materials, experimental systems and methods used in many studies. Here, indicate whether each material, system or method listed is relevant to your study. If you are not sure if a list item applies to your research, read the appropriate section before selecting a response.

Materials & experimental systems

n/a	Involved in the study	
☐	☒	Antibodies
☐	☒	Eukaryotic cell lines
☒	☐	Palaeontology and archaeology
☐	☒	Animals and other organisms
☒	☐	Human research participants
☒	☐	Clinical data
☒	☐	Dual use research of concern

Methods

n/a	Involved in the study	
☒	☐	ChIP-seq
☒	☐	Flow cytometry
☒	☐	MRI-based neuroimaging

Antibodies

Antibodies used	Alexa-647 conjugated M1 antibody (1:1000): mouse M1 monoclonal anti-FLAG (Sigma, cat# 3040) in-house conjugated to Alexa-647;<br>chicken anti-GFP (1:500; Abcam, cat# ab13970);<br>rabbit anti-mCherry (1:500; Abcam, cat# ab183628);<br>mouse anti-orexin-A (1:500; Santa Cruz Biotechnology, cat# sc80263);<br>Alexa-488-labeled goat anti-chicken (1:1000, Abcam, cat# ab150169);<br>Alexa-488-labeled goat anti-mouse (1:1000, Thermo Fisher, cat# A32723);

	Alexa-546-labeled goat anti-rabbit (1:1000, Thermo Fisher, cat# A11035);<br>Alexa-568-labeled goat anti-mouse (1:500, Thermo Fisher, cat# A11004);
Validation	Alexa-647 conjugated M1 antibody was validated for live-cell labeling by the von Zastrow laboratory (UCSF) [Irannejad, R. et al. Nature 495, 534-538 (2013)]. The chicken anti-GFP antibody was validated by Iwasaki S. and Ikegaya Y, Cereb Cortex 31, 785-794 (2021). The rabbit anti-mCherry antibody was validated by Li L., et al., Cell Death Dis 12:220 (2021). The mouse anti-orexin antibody was validated by Zhan, S. et al, J Cell Mol Med 23, 6822-6835 (2019). All secondary antibodies were validated by the commercial provider.

Eukaryotic cell lines

Policy information about cell lines

Cell line source(s)	HEK293T cells (ATCC cat#3216)
Authentication	The cell lines were authenticated by the vendor (ATCC) using Short Tandem Repeat (STR) Profiling to detect misidentified, cross-contaminated, or genetically-drifted lines.
Mycoplasma contamination	The cell line used was mycoplasma-free.
Commonly misidentified lines<br>(See ICLAC register)	The study did not involve commonly misidentified cell lines.

Animals and other organisms

Policy information about studies involving animals; ARRIVE guidelines recommended for reporting animal research

Laboratory animals	To prepare neuronal cultures, E17 rat embryos obtained from timed-pregnant female Wistar rats were used in this study. For ex vivo and in vivo experiments, 4-24 week old mice (mus musculus, C57/Bl6 strain) of both sexes were used. Animals were maintained under normal at room temperature and humidity levels.
Wild animals	No wild animals were used in this study.
Field-collected samples	No field-collected samples were used in this study.
Ethics oversight	Animal procedures were performed in accordance to the guidelines of the European Community Council Directive or the Animal Welfare Ordinance (TSchV 455.1) of the Swiss Federal Food Safety and Veterinary Office and were approved by the Zürich or Bern Cantonal Veterinary Office, the government of Upper Bavaria, or the National Council on Animal Care of the Italian Ministry of Health.

Note that full information on the approval of the study protocol must also be provided in the manuscript.
